# The CALIPR framework for highly accelerated myelin water imaging with improved precision and sensitivity

**DOI:** 10.1126/sciadv.adh9853

**Published:** 2023-11-01

**Authors:** Adam V. Dvorak, Dushyant Kumar, Jing Zhang, Guillaume Gilbert, Sharada Balaji, Neale Wiley, Cornelia Laule, G.R. Wayne Moore, Alex L. MacKay, Shannon H. Kolind

**Affiliations:** ^1^ Physics and Astronomy, University of British Columbia, Vancouver, BC, Canada.; ^2^ International Collaboration on Repair Discoveries, University of British Columbia, Vancouver, BC, Canada.; ^3^ Radiology, University of Pennsylvania, Philadelphia, PA, USA.; ^4^ Global MR Applications & Workflow, GE HealthCare Canada, Mississauga, ON, Canada.; ^5^ MR Clinical Science, Philips Canada, Mississauga, ON, Canada.; ^6^ Radiology, University of British Columbia, Vancouver, BC, Canada.; ^7^ Pathology and Laboratory Medicine, University of British Columbia, Vancouver, BC, Canada.; ^8^ UBC MRI Research Centre, University of British Columbia, Vancouver, BC, Canada.; ^9^ Medicine (Neurology), University of British Columbia, Vancouver, BC, Canada.

## Abstract

Quantitative magnetic resonance imaging (MRI) techniques are powerful tools for the study of human tissue, but, in practice, their utility has been limited by lengthy acquisition times. Here, we introduce the Constrained, Adaptive, Low-dimensional, Intrinsically Precise Reconstruction (CALIPR) framework in the context of myelin water imaging (MWI); a quantitative MRI technique generally regarded as the most rigorous approach for noninvasive, in vivo measurement of myelin content. The CALIPR framework exploits data redundancy to recover high-quality images from a small fraction of an imaging dataset, which allowed MWI to be acquired with a previously unattainable sequence (fully sampled acquisition 2 hours:57 min:20 s) in 7 min:26 s (4.2% of the dataset, acceleration factor 23.9). CALIPR quantitative metrics had excellent precision (myelin water fraction mean coefficient of variation 3.2% for the brain and 3.0% for the spinal cord) and markedly increased sensitivity to demyelinating disease pathology compared to a current, widely used technique. The CALIPR framework facilitates drastically improved MWI and could be similarly transformative for other quantitative MRI applications.

## Teaser

The CALIPR framework can drastically reduce acquisition time for quantitative MRI while simultaneously improving the data quality.

## INTRODUCTION

Quantitative magnetic resonance imaging (MRI) techniques can provide objective measures of tissue microstructure properties, but their clinical utility tends to be limited by lengthy acquisition times due to sampling a tissue parameter mapping dimension in addition to the spatial image dimensions. As a result, data are often acquired with relatively low image resolution or using acceleration methods that reduce image quality, which limits the sensitivity of the resulting quantitative metrics. Faster semiquantitative techniques can be acquired instead, but these tend not to provide the same degree of reproducibility or specificity to tissue properties of interest. An approach for drastically accelerating quantitative MRI acquisition, while retaining sensitivity and specificity to tissue properties, would be of tremendous scientific and clinical value.

Multicomponent T_2_ mapping is a class of quantitative MRI techniques, which characterize tissue by identifying MRI signal contributions from multiple water pools, each with a distinct range of T_2_ relaxation times. The most prolific multicomponent T_2_ mapping technique, myelin water imaging (MWI) (*1*), has proven valuable for the study of development, aging, disease, injury, genetics, and fundamental biology in the central nervous system (*2*–*9*). MWI can characterize signal from water trapped between myelin lipid bilayers [T_2_ < 40 ms in normal white matter (WM) at 3 tesla] and from water in intra- and extracellular spaces (40 ms < T_2_ < 200 ms) (*1*). Using MWI data, the total fraction of signal from short T_2_ myelin water [myelin water fraction (MWF)] and the geometric mean T_2_ of intra- and extracellular water (IET2) can be calculated. MWF correlates strongly with quantitative histopathologic measures of myelin density (*4*, *10*), and IET2 also shows clinical relevance (*11*).

Multicomponent T_2_ mapping techniques have also proven valuable for application to a wide variety of other human tissues, such as in muscle for the study of diabetes and neuromuscular disease activity, or in prostate for noninvasively identifying and differentiating grades of cancer tissue in prostate (*12*–*15*).

However, multicomponent T_2_ mapping techniques involve acquisition of many separate T_2_-weighted (T_2_w) images, on the order of 30 to 50 for MWI, which necessitates extremely lengthy data collection times using existing MRI acquisition and reconstruction approaches.

Previous work accelerating MWI has drastically reduced acquisition time from 26 min to acquire a single two-dimensional (2D) slice (*1*) to approximately 10 to 20 min for full brain coverage (*16*–*19*). A very common MWI acceleration approach is the use of the gradient and spin-echo (GRASE) pulse sequence, which uses additional gradient echoes to acquire three times the data in the same amount of time. The GRASE sequence is also often coupled with additional acceleration from under sampling, usually in the form of parallel imaging, providing a total acceleration factor of approximately 6 to 12. However, GRASE suffers from blurring induced by the lower signal-to-noise ratio (SNR) gradient echoes and increased minimum echo spacing (ΔTE), sensitivity to motion, peripheral nerve stimulation (PNS), acoustic noise, and possible orientation effects (*20*, *21*). Furthermore, GRASE is highly dependent on the MRI scanner gradient performance, making it more sensitive to gradient instabilities and limiting availability between scanners with different specifications.

Compressed sensing (CS) is an increasingly common acceleration approach (*22*, *23*) that was recently used to reduce MWI acquisition time by a factor of 10 using under sampling acceleration alone (*19*). Although it avoids the issues associated with GRASE, this acceleration approach still requires data to be acquired with relatively large voxel volumes (~10 mm^3^) to achieve acquisition times under 10 min. Furthermore, some blurring and loss of effective resolution can be introduced due to the limited ability to suppress under sampling artifacts when implementing a standard CS framework that exploits only the spatial dimensions of the acquisition and does not include the parameter mapping dimension.

Regardless of the acceleration approach, current MWI techniques tend to acquire data at relatively low resolution to keep total acquisition times near 10 min, usually with highly nonisotropic voxels and total voxel volumes of about 10 mm^3^. To address these limitations, in this work we introduce, validate, and assess a novel comprehensive framework for drastically accelerating quantitative MRI, referred to as the Constrained, Adaptive, Low-dimensional, Intrinsically Precise Reconstruction (CALIPR) framework.

The CALIPR framework was motivated by a series of recent advancements in MRI. It has been demonstrated that the principles of CS can be extended to exploit additional parameter mapping dimensions and facilitate higher under sampling acceleration factors (*24*). Furthermore, pivotal work by Huang *et al.*, Tamir *et al.*, and others (*25*–*29*) demonstrated that image reconstruction can be constrained to a subspace of these additional nonspatial dimensions to improve performance. For example, subspace constrained reconstruction for a fast spin-echo sequence allows information to be shared throughout the echo train while accounting for signal evolution in the reconstruction process. In alternative methods where this signal evolution is not accounted for, such as the GRASE sequence (*30*, *31*) or fast spin-echo echo sharing (*32*), image blurring is caused by the broken Fourier relationship between *k*-space and image space. Recently, principal components analysis (PCA) denoising has been shown to be highly effective at reducing errors in quantitative MRI metrics, including those from diffusion (*33*) and multicomponent T_2_ mapping techniques (*34*). If PCA denoising was incorporated directly into image reconstruction, it could be used to exploit data redundancy even more effectively than when denoising already reconstructed images. Building upon these concepts, CALIPR is an extended CS framework, which exploits redundancy present in the data by reconstructing images that are constrained to lie within a low-dimensional PCA subspace.

## RESULTS

Details of the CALIPR framework itself are described in Materials and Methods. The experiments described below fall into three sections. First, we validated the theoretical benefits of the CALIPR framework by retrospectively under sampling a post-mortem fixed brain MWI dataset, showing improved performance compared to the currently adopted CS acceleration technique. Second, after implementing CALIPR for in vivo brain and spinal cord MWI, we demonstrated that the resulting quantitative maps have excellent reproducibility despite being acquired with very high under sampling acceleration factors. Last, we showed that the intrinsic denoising and high resolution of CALIPR provide increased sensitivity to demyelinating disease pathology, particularly for MWF, compared to a commonly used GRASE MWI approach.

### Validation with retrospective under sampling

**Fig. 1.**
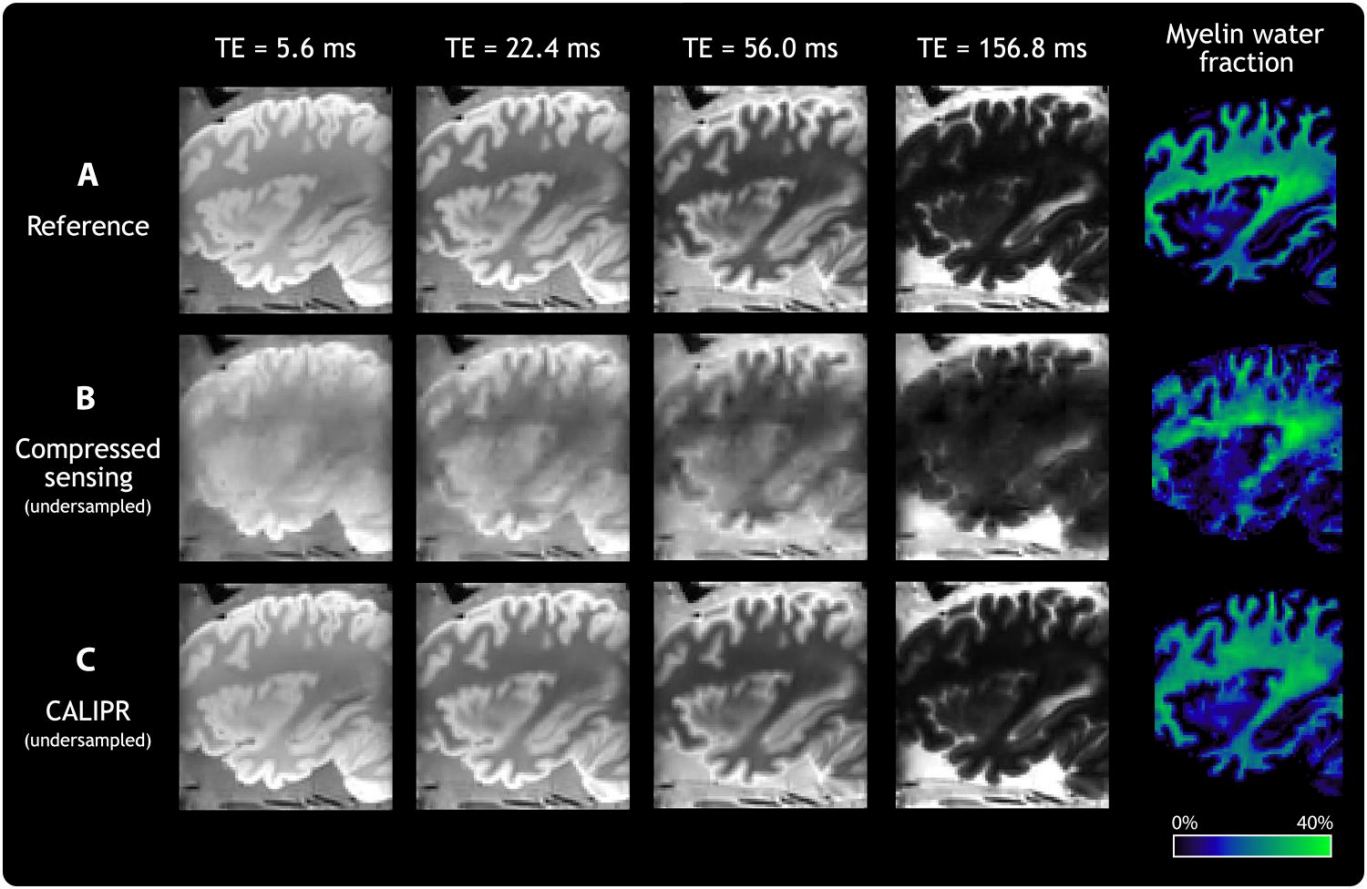
Retrospective under sampling validation. Echo time (TE) images and quantitative MWF maps for a MWI dataset acquired in a post-mortem, single-hemisphere fixed brain sample. (**A**) Reference version, which reconstructed the entire acquired dataset with a CS reconstruction. (**B**) Retrospectively under sampled CS version, which used an acceleration factor of 14.6 (6.8% of the dataset). (**C**) Retrospectively under sampled CALIPR version, which used the same acceleration factor as (B) but with the additional CALIPR adaptive subspace constraint.

Figure 1A shows results from the full reference acquisition dataset acquired in fixed brain. CS and CALIPR reconstructions, shown in Fig. 1, B and C, respectively, were performed after retrospectively under sampling the dataset with acceleration factor 14.6 (6.8% of the dataset). CALIPR echo images and MWF maps demonstrated markedly improved suppression of under sampling artifacts compared to CS. As the only difference between the CS and CALIPR iterative reconstructions was the subspace constraint incorporated into CALIPR, this comparison directly demonstrates the efficacy of enforcing a low-dimensional representation of the signal along the parameter mapping dimension.

### Reproducibility in healthy brain and spinal cord

We developed CALIPR acquisitions for the brain and spinal cord MWI. Reproducibility was assessed by performing each acquisition twice, in separate exams with repositioning, for five healthy participants. Further details are provided in Materials and Methods.

**Fig. 2.**
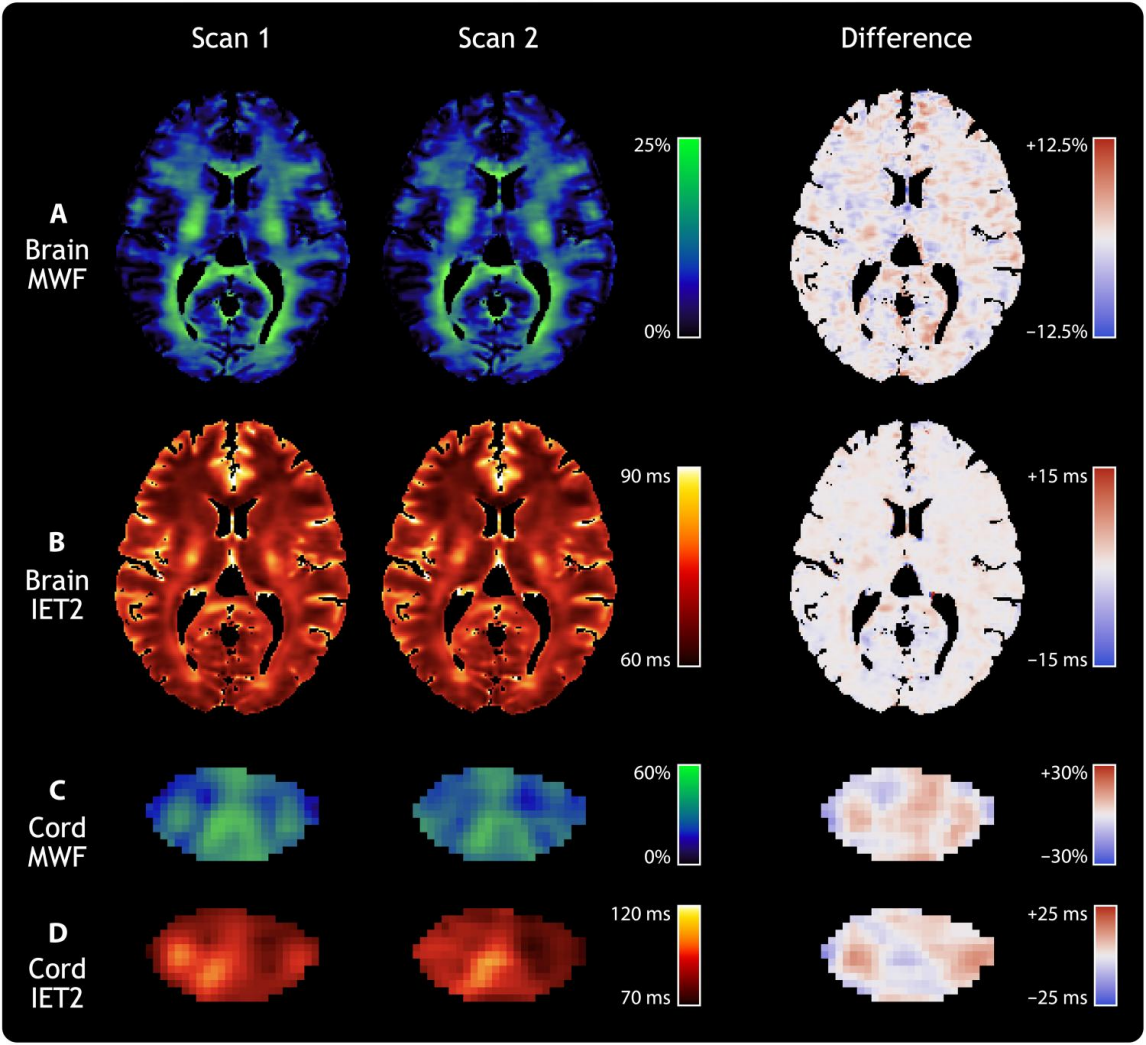
Reproducibility of MWI metrics in brain and spinal cord. Individual slices of the (**A**) brain MWF, (**B**) brain geometric mean of IET2, (**C**) spinal cord MWF, and (**D**) spinal cord IET2 for a single healthy participant. Columns show results from two separate CALIPR MWI exams, aligned in the subject’s structural image space (T_1_-weighted for the brain and T_2_*-weighted for the cord), along with their voxel-wise difference maps.

Figure 2 shows individual slices of CALIPR quantitative maps from each exam, along with voxel-wise difference maps, for one of the reproducibility subjects. The reproducibility results showed excellent qualitative agreement between exams, with limited magnitude and spatial extent of differences.

**Fig. 3.**
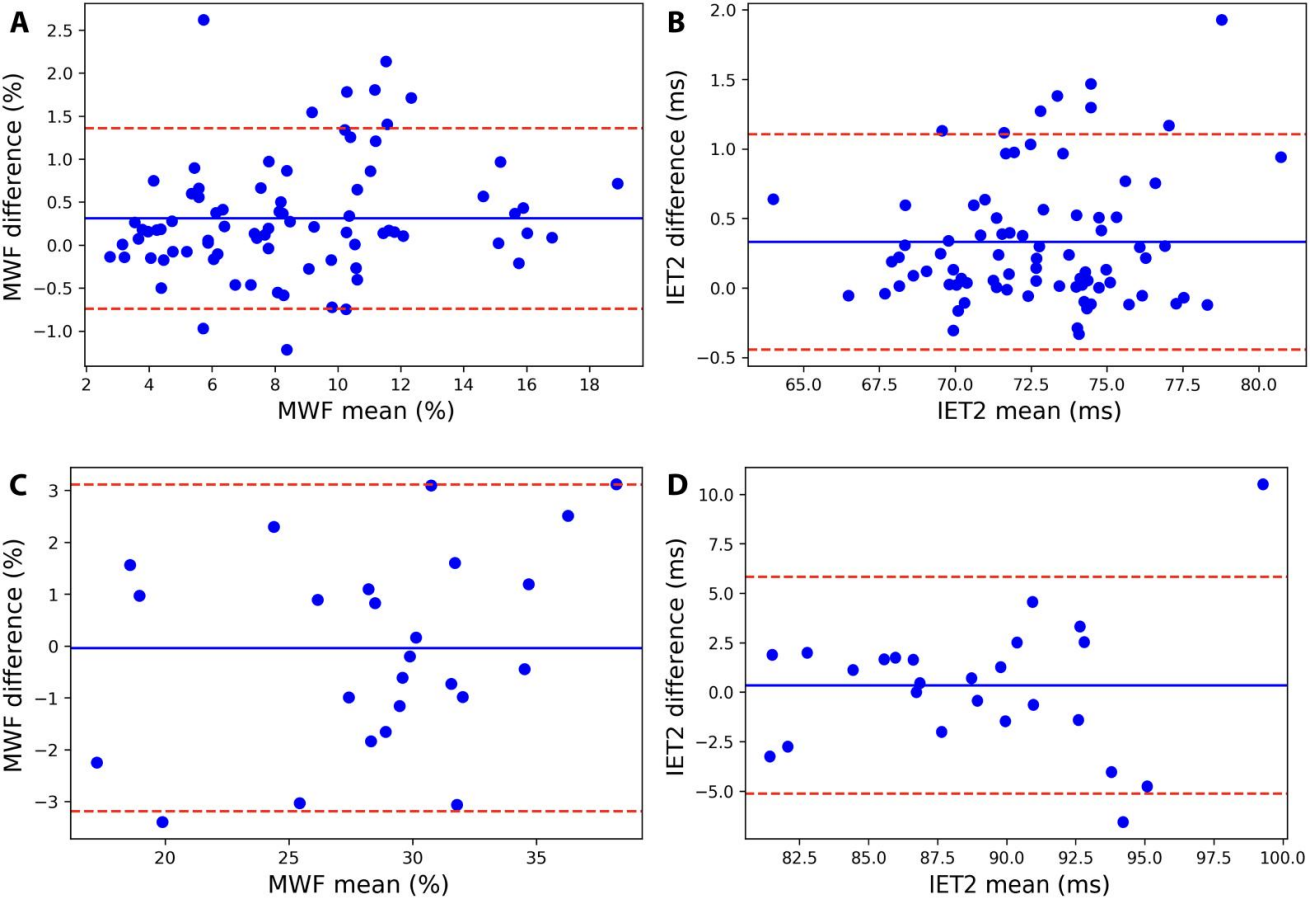
Bland-Altman plots. Bland-Altman plots for CALIPR reproducibility subjects’ (**A**) brain MWF, (**B**) brain geometric mean of IET2, (**C**) spinal cord MWF, and (**D**) spinal cord IET2 ROI results from separate exams. Mean values from both exams are plotted on the *x* axis, and differences are plotted on the *y* axis. Solid lines indicate the mean bias (difference) of all data points [(A) +0.3%, (B) +0.3 ms, (C) +0.0%, and (D) +0.4 ms). Dotted lines indicate the positive (+1.96 SD) and negative (−1.96 SD) 95% limits of agreement. Brain ROIs include all WM, all gray matter (GM), and combined WM and GM from T_1_w image segmentations, along with nine additional WM ROIs [all JHU WM labels combined: genu of corpus callosum (CC); splenium of CC; whole CC; posterior internal capsule; and frontal, occipital, parietal, and temporal lobes masked to WM] and four additional GM ROIs (cortical GM, caudate, thalamus, and putamen). Spinal cord ROIs include the whole cord, WM, GM, dorsal column, and lateral corticospinal tracts.

Agreement between exams is further visualized with Bland-Altman plots in Fig. 3, which do not show evidence of a significant systematic bias (mean bias, Fig. 3A brain MWF: +0.3%, Fig. 3B brain IET2: +0.3 ms, Fig. 3C spinal cord MWF: 0.0%, and Fig. 3D spinal cord IET2: +0.4 ms).

CALIPR MWF region of interest (ROI) results and corresponding reproducibility metrics are summarized in Table 1 for the brain and in Table 2 for the spinal cord. MWF mean repeatability coefficient (RC), coefficient of variation (COV), and intraclass correlation coefficient (ICC) were 0.7, 3.2%, and 0.92 for the brain and 2.2, 3.0%, and 0.86 for the spinal cord.

**Table 1.** CALIPR brain MWF reproducibility. Reproducibility results for the brain myelin water fraction (MWF) from repeated CALIPR scans, acquired in separate exams, for five healthy participants. Mean and SD of the region of interest (ROI) values are shown along with the following reproducibility metrics: the 95% limits of agreement (LOAs) (LOA negative|LOA positive), repeatability coefficients (RC), coefficients of variation (COVs), and intraclass correlation coefficients (ICCs). Brain ROIs include all WM, all gray matter (GM), and combined WM and GM from T_1_-weighted image segmentations, along with nine additional WM ROIs [all JHU WM labels combined: genu of corpus callosum (CC); splenium of CC; whole CC; posterior internal capsule; and frontal, occipital, parietal, and temporal lobes masked to WM] and four additional GM ROIs (cortical GM, caudate, thalamus, and putamen).

		CALIPR MWF (*%*)					
	ROI	Exam 1	Exam 2	LOA	RC	COV (%)	ICC
	WM and GM	6.4 ± 0.8	6.3 ± 0.8	−0.3 | 0.4	0.2	1.3	0.99
WM regions	WM	8.8 ± 1.4	8.7 ± 1.4	−0.7 | 1.1	0.6	2.4	0.97
	All JHU	11.6 ± 1.8	11.0 ± 2.1	−1.0 | 2.1	1.0	3.3	0.94
	Genu	11.9 ± 2.4	11.5 ± 2.3	−1.2 | 2.0	1.0	3.3	0.96
	Splenium	13.2 ± 1.9	12.3 ± 2.3	−0.6 | 2.6	1.4	4.2	0.92
	Whole CC	12.0 ± 1.9	11.4 ± 2.3	−1.0 | 2.3	1.2	4.0	0.94
	Posterior Internal Capsule	16.3 ± 1.5	15.7 ± 1.5	0.2 | 1.0	0.8	1.9	0.96
Lobe WM	Frontal	7.3 ± 1.2	7.5 ± 1.0	−0.7 | 0.5	0.4	2.1	0.98
	Occipital	9.1 ± 1.5	9.0 ± 1.8	−0.8 | 1.1	0.6	2.5	0.98
	Parietal	8.7 ± 1.6	8.5 ± 1.6	−0.7 | 1.2	0.6	2.7	0.97
	Temporal	6.5 ± 1.1	6.1 ± 1.2	−0.1 | 1.0	0.6	3.8	0.95
GM regions	GM	3.9 ± 0.5	3.9 ± 0.4	−0.3 | 0.3	0.2	1.8	0.97
	Cortical	3.5 ± 0.5	3.4 ± 0.4	−0.3 | 0.4	0.2	2.1	0.97
	Caudate	5.8 ± 0.7	5.3 ± 0.7	−1.8 | 2.8	1.3	8.1	0.60
	Thalamus	9.5 ± 1.1	9.5 ± 0.8	−1.7 | 1.8	0.9	3.8	0.82
	Putamen	4.7 ± 0.6	4.6 ± 0.5	−0.7 | 1.1	0.6	4.5	0.84
Mean		8.7 ± 1.3	8.4 ± 1.3	−0.7 | 1.4	0.7	3.2	0.92

**Table 2.** CALIPR spinal cord MWF reproducibility. Reproducibility results for the spinal cord MWF from repeated CALIPR scans, acquired in separate exams, for 5 healthy participants. Mean and SD of the ROI values are shown along with the following reproducibility metrics: the 95% LOAs (LOA negative|LOA positive), RCs, COVs, and ICCs. Spinal cord ROIs include the whole cord (WC), WM, GM, dorsal column (DC), and lateral corticospinal tracts (LCSTs).

	CALIPR MWF (*%*)					
ROI	Exam 1	Exam 2	LOA	RC	COV (%)	ICC
WC	28.0 ± 1.1	28.3 ± 1.5	−2.3 | 1.7	1.4	1.8	0.85
WM	30.1 ± 1.1	30.4 ± 1.7	−2.0 | 1.3	1.1	1.4	0.91
GM	19.7 ± 3.1	19.9 ± 2.1	−4.6 | 4.2	2.9	5.3	0.84
DC	35.3 ± 3.4	34.1 ± 2.2	−1.5 | 4.0	2.1	2.0	0.91
LCST	29.3 ± 3.2	29.9 ± 2.1	−5.6 | 4.3	3.5	4.3	0.78
Mean	28.5 ± 2.4	28.5 ± 1.9	−3.2 | 3.1	2.2	3.0	0.86

For CALIPR IET2, reproducibility is summarized in table S1 for the brain and table S2 for the spinal cord. IET2 mean RC, COV, and ICC were 0.05, 0.26%, and 0.98 for the brain and 0.35, 1.4%, and 0.74 for the spinal cord.

Comparing MWF values from exams 1 and 2, a small significant bias was found in the posterior internal capsule (two-sided one-sample *t* test; mean bias, +0.6%, *P* = 0.005) and in temporal WM (+0.4%, *P* = 0.04). There was no significant difference in IET2 values for any of the 16 brain or 5 spinal cord ROIs.

### Sensitivity to demyelinating disease pathology

**Fig. 4.**
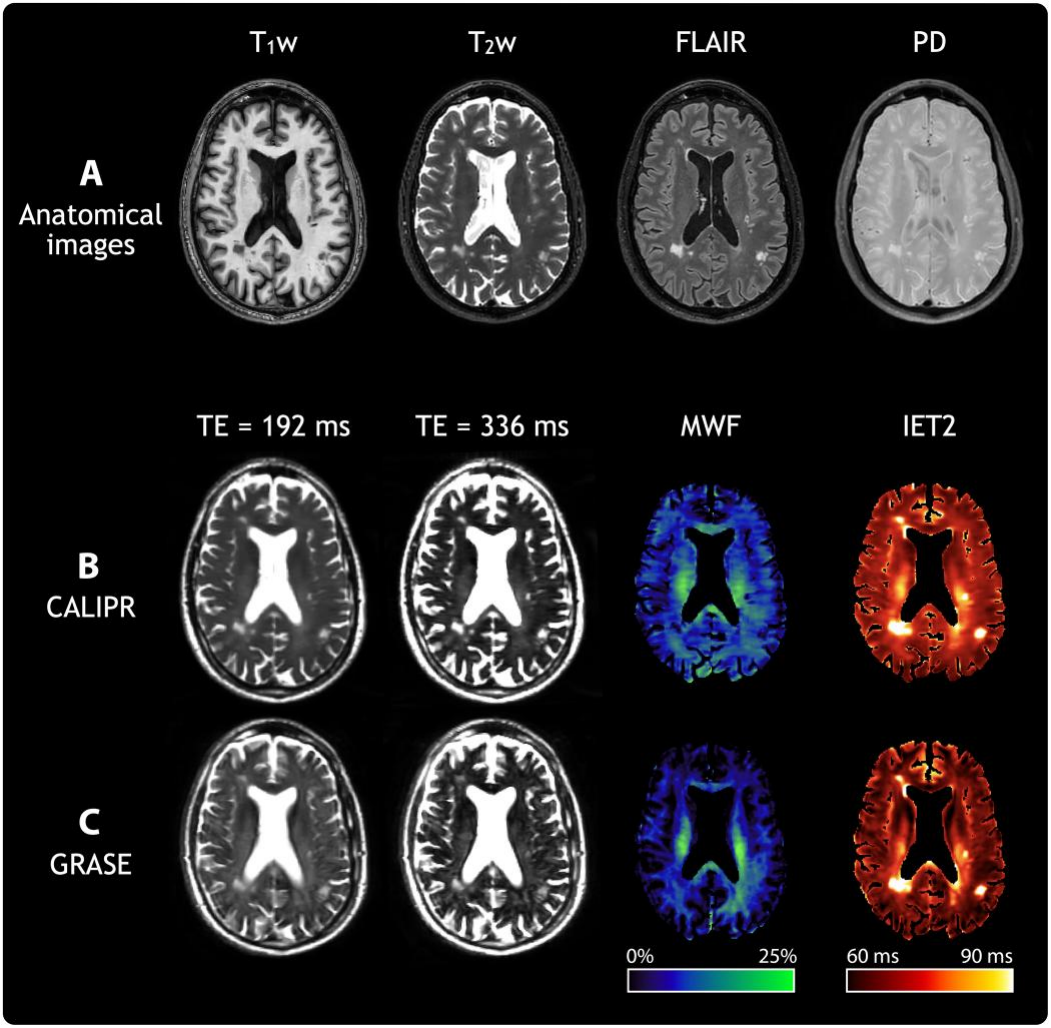
Anatomical and MWI results for a subject living with multiple sclerosis. A representative image slice from anatomical and MWI results in brain for a subject living with relapsing-remitting multiple sclerosis (male, age 60 years, expanded disability status scale of 2.0, and disease duration of 12 years). (**A**) From left to right, the columns show T_1_-weighted (T_1_w), T_2_w, fluid-attenuated inversion recovery (FLAIR), and proton density (PD) anatomical images. For (**B**), our proposed CALIPR MWI approach and (**C**) a commonly used GRASE MWI approach, from left to right the columns, show two MWI images at different echo times (TE) as well as quantitative MWF and geometric mean of IET2 maps. The same slice is shown for all images and maps, which were aligned in the subject’s T_1_w image space.

To assess sensitivity to pathological tissue changes, we acquired data from a subject living with relapsing-remitting multiple sclerosis [male, age 60 years, expanded disability status scale of 2.0 (*35*), and disease duration of 12 years] using CALIPR and with the commonly used GRASE MWI approach for comparison. Figure 4 shows conventional anatomical imaging (Fig. 4A), CALIPR MWI (Fig. 4B), and GRASE MWI (Fig. 4C) results for a single axial slice with multiple focal lesions.

Compared to GRASE, the CALIPR image at echo time (TE) of 192 ms shows sharper delineation of anatomical structure borders, such as the ventricles and cortical gyri. The CALIPR images also show less evidence of artifacts related to under sampling or noise. This denoising effect is especially evident for long TE images, such as TE of 336 ms, which have lower SNR due to T_2_ decay.

**Fig. 5.**
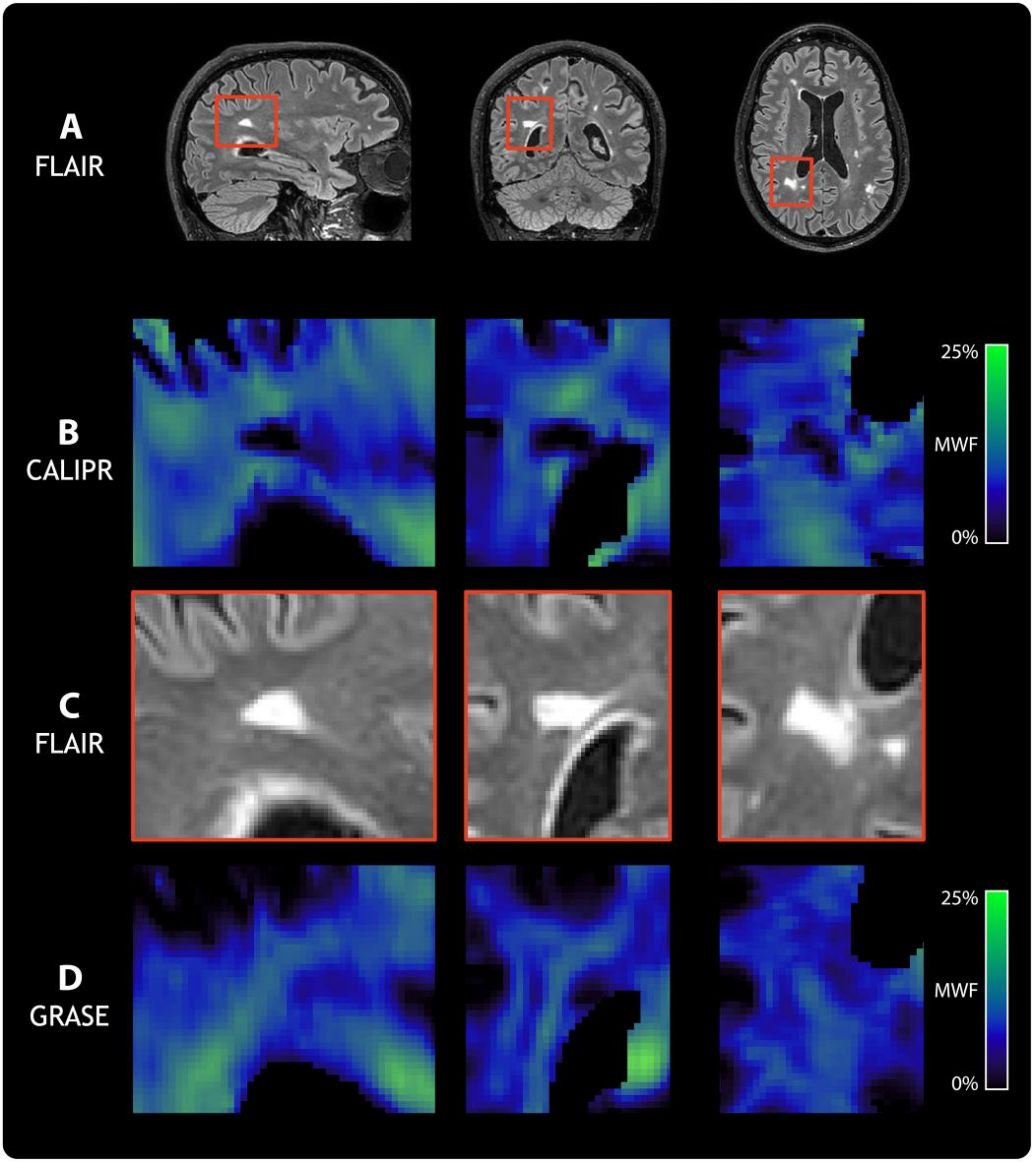
Comparison of MWF sensitivity to demyelinating disease pathology. From left to right, columns show sagittal, coronal, and axial orientation views, respectively, for a subject living with relapsing-remitting multiple sclerosis (male, age 60 years, expanded disability status scale of 2.0, and disease duration of 12 years). (**A**) Full slices of a FLAIR anatomical image are shown with red boxes highlighting a region centered around a particularly large FLAIR hyperintense lesion. Close-up views of this region are shown in (**B**) for MWF from our proposed CALIPR MWI approach, in (**C**) for FLAIR, and in (**D**) for MWF from a commonly used GRASE MWI approach. All images and maps were aligned in the subject’s T_1_-weighted image space to ensure that the exact same region could be compared.

**Fig. 6.**
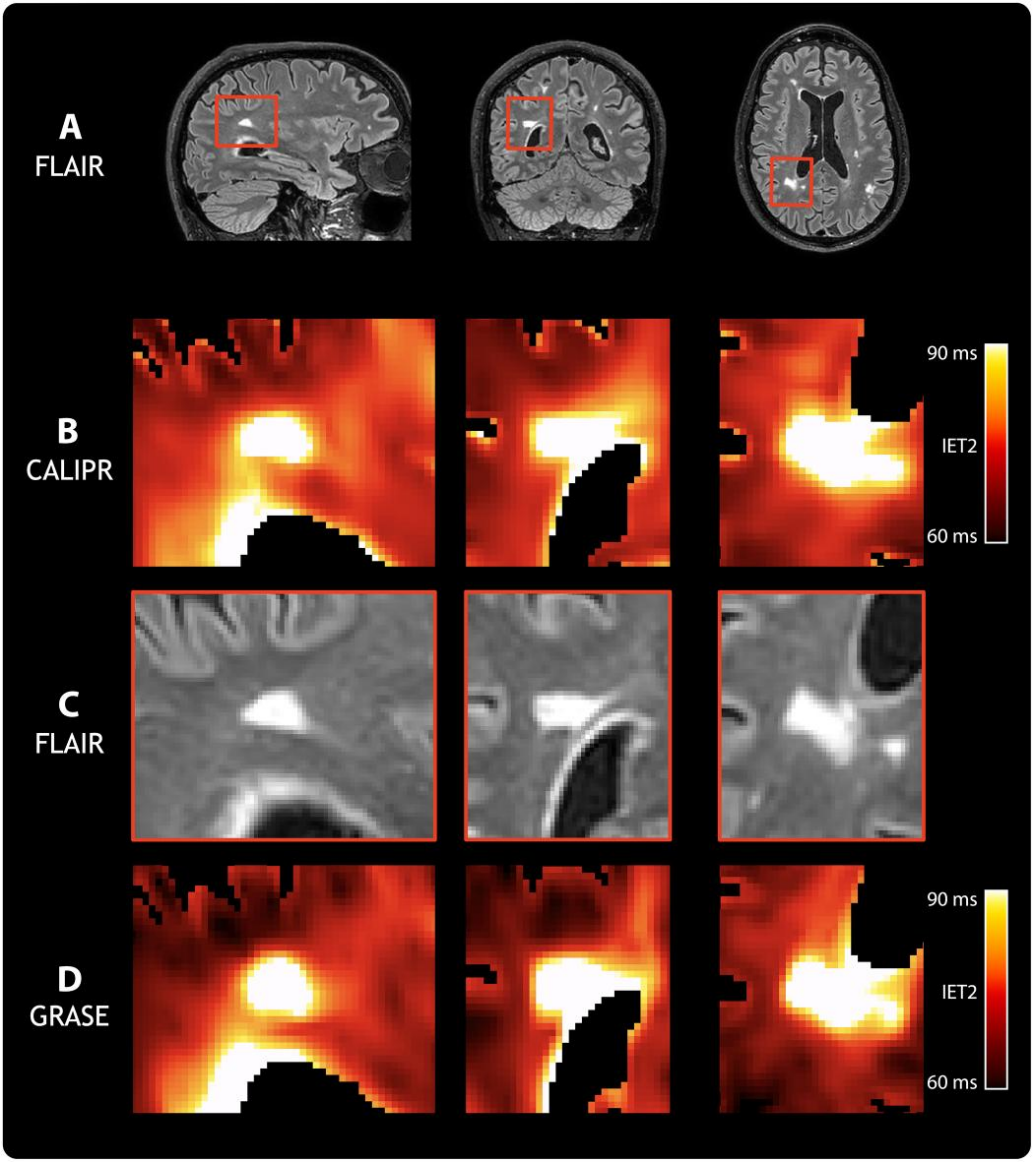
Comparison of geometric mean of IET2 sensitivity to demyelinating disease pathology. From left to right, columns show sagittal, coronal, and axial orientation views, respectively, for a subject living with relapsing-remitting multiple sclerosis (male, age 60 years, expanded disability status scale of 2.0, and disease duration of 12 years). (**A**) Full slices of a FLAIR anatomical image are shown with red boxes highlighting a region centered around a particularly large FLAIR hyperintense lesion. Close-up views of this region are shown in (**B**) for IET2 from our proposed CALIPR MWI approach, in (**C**) for FLAIR, and in (**D**) for IET2 from a commonly used GRASE MWI approach. All images and maps were aligned in the subject’s T_1_-weighted image space to ensure that the exact same region could be compared.

Multiple sclerosis lesions appeared more conspicuous in CALIPR echo images compared to GRASE. The CALIPR echo images showed comparable quality to the conventional T_2_w anatomical image and even improved visualization of T_2_ hyperintense lesions at very late echo times, where healthy brain tissue has almost no signal remaining. In Figs. 5 and 6, sagittal, coronal, and axial fluid-attenuated inversion recovery (FLAIR) image slices are shown (Figs. 5A and 6A) along with close-up views of a region centered around a large lesion (Figs. 5C and 6C).

For MWF in Fig. 5, CALIPR showed increased sensitivity to the signal changes expected in a demyelinating disease lesion (Fig. 5B) and much sharper congruence between lesion tissue visible on FLAIR and low MWF values compared to GRASE (Fig. 5D). In Fig. 6, CALIPR and GRASE show relatively similar evidence of increased IET2 within lesions.

## DISCUSSION

We have demonstrated the utility of CALIPR as a comprehensive framework for rapid acquisition of precise, sensitive multicomponent quantitative T_2_ relaxation data. Our validation experiments demonstrated vastly improved performance of CALIPR compared to conventional CS, one of the main methods currently used for MWI (*19*). This result was expected, as the CALIPR framework extends the principles of CS (incoherent sampling acquisition and sparse representation reconstruction) to the additional parameter mapping dimension intrinsic to quantitative MRI. Unlike most previous MWI techniques, where the same *k*-space data points are acquired throughout the echo train, the CALIPR framework extends the principle of incoherent sampling by acquiring a different pseudo-random *k*-space sampling scheme for each TE. On the reconstruction side, instead of reconstructing data from each TE individually, the CALIPR framework reconstructs data from the entire echo train together, which allows information to be shared between TEs to better inform the reconstruction. This joint reconstruction also allows the CS principle of sparse representation to be extended, by explicitly constraining the reconstruction to a subspace of the principal signal evolution components.

The acquisition and reconstruction work in tandem to improve performance using these CS principles. The acquisition sampling results in noise-like under sampling artifacts, which are incoherent along the parameter mapping dimension (they differ between TEs), meaning that they are intrinsically nonsparse and will be suppressed by the sparsity-promoting reconstruction. Alternatively, true image features are coherent along the parameter mapping dimension (they are strongly correlated between TEs) and can therefore be sparsely represented and easily recovered during reconstruction.

CALIPR MWI metrics had excellent reproducibility in both the brain and spinal cord, with improved reproducibility metrics compared to previous techniques, despite the use of much higher under sampling acceleration factors. Our mean brain MWF RC of 0.7 is better than those previously reported for MWI using 2D multislice GRASE (2.1 with parallel imaging under sampling acceleration factor of 2.0) (*18*) or 3D multi-echo spin echo (MESE) with conventional CS (1.5 with under sampling acceleration factor of 10.0) (*19*). Our mean brain MWF COV of 3.2% compares favorably with those previously reported for MWI using single-slice 2D MESE (19%, 12.6%) (*36*, *37*), 3D MESE (4.0%) (*38*), 3D GRASE (13.4% for a single subject) (*39*), 2D multislice GRASE (6.7% with parallel imaging under sampling acceleration factor of 2.0) (*18*), and 3D MESE with conventional CS (6.2% with acceleration factor 10.0) (*19*). Our mean brain MWF ICC of 0.92 is better than those previously reported for MWI using 3D MSE (intrasite 0.76) (*38*), 3D GRASE (0.83) (*40*), 2D multislice GRASE (0.80 with parallel imaging under sampling acceleration factor of 2.0) (*18*), or 3D MESE with conventional CS (0.79 with under sampling acceleration factor of 10) (*19*). For the spinal cord, our mean MWF COV values in whole cord (WC), gray matter (GM), dorsal column (DC), and lateral corticospinal tracts (LCSTs) (1.8, 5.3, 2.0, and 3.4%, respectively) were lower than those previously reported for 3D GRASE with parallel imaging under sampling acceleration factor 2.0 in the same ROIs (6.1, 11.5, 7.7, and 8.1%, respectively) (*41*). The improved reproducibility of CALIPR MWI is extremely encouraging for use in longitudinal studies, which have often been hindered by a limited sensitivity to detect small changes over time (*42*).

Although MWF absolute values vary somewhat with sequence parameters, such as echo spacing or repetition time (TR), we can compare the rank of MWF values between notable brain and spinal cord ROIs to previous work. MWF values reported in literature show a clear trend of decreasing brain MWF between the posterior internal capsule, splenium of the corpus callosum (CC), genu of the CC, all WM, and all GM, as well as decreasing spinal cord MWF between the DC, WM, LCST, and GM (*43*). Previous MWI atlases have reported mean WM/GM MWF contrast ratios of 2.0 in the brain and 1.5 in the spinal cord, in good agreement with the values found here (2.0 in the brain and 1.5 in the spinal cord), despite our relatively small sample size and demographic range compared to atlas-based studies (*43*).

Agreement of the ranking of MWF values between ROIs and of the MWF contrast between tissue types bolsters our confidence in the validity of our results, especially when noting the heterogeneity of MWF values within WM. In particular, post-mortem studies have identified a gradient in myelin content between the splenium and genu of the CC (*44*), which is identifiable in the results presented here and the wider MWI literature, but is often not reflected in the metrics produced by alternative myelin-sensitive MRI techniques (*43*).

We demonstrated markedly improved sensitivity to demyelinating disease pathology using CALIPR compared to GRASE, which is likely due to a combination of factors. We implemented CALIPR with a relatively simple 3D MESE sequence, which provided several advantages. When comparing the GRASE and MESE sequences, the additional echo-planar imaging gradient echo readouts used by GRASE increase the minimum echo spacing, which reduces image SNR due to T_2_ decay and reduces the ability to detect and characterize the short T_2_ myelin water signal. GRASE also introduces image blurring dependant on T_2_* because high-frequency *k*-space is acquired with additional T_2_* weighting (not pure T_2_). Compared to GRASE, the MESE sequence allowed a larger number of echoes to be sampled (56 instead of 48 for GRASE) using shorter echo spacing (6.0 ms instead of 8.0 ms for GRASE).

Furthermore, the CALIPR framework links the spatial and parameter mapping dimensions, effectively spreading under sampling between both, so that the higher number of echoes (larger parameter mapping dimension) can be leveraged to facilitate higher under sampling acceleration factors. This allowed the CALIPR sequence to be acquired with >3× the total acceleration of GRASE (23.9 versus 7.5), which can be leveraged to acquire data with the same resolution in a fraction of the time or to acquire much higher-resolution data in the same acquisition time.

The relative simplicity of the MESE sequence is also perfectly suited to generating sampling schemes, which are maximally incoherent: A substantial contributing factor to the strong performance of the CALIPR framework. In comparison, the GRASE sequence has a high degree of intrinsic sampling coherence introduced by the echo-planar imaging readouts. Although the GRASE sequence is valuable for many MRI applications, this limitation precludes much of the potential benefits of attempting to use incoherent under sampling pattern and generally encourages GRASE to be paired with more conventional parallel imaging-based methods.

In combination, the shorter echo spacing and increased acceleration of CALIPR improve its ability to resolve signal components in the parametric dimension and anatomical features in the spatial dimensions.

Furthermore, the lower PNS and acoustic noise of the MESE sequence, compared to GRASE, makes it available for more subject groups, including those whose ability to undergo MRI is conditional (such as people with metallic implants) or populations that are particularly sensitive to noise (such as pediatric subjects). For example, even the high-resolution CALIPR brain MWI sequence studied in our reproducibility experiments can be operated in normal PNS mode (<80% of the limit), while the previously presented GRASE sequence cannot (94% of PNS limit).

For our implementation of CALIPR MWI for the spinal cord, in addition to the brain, very few modifications were required to adapt the sampling scheme and reconstruction code. Sampling schemes were all generated using the same pattern, differing primarily by matrix size to match the acquisition. Iterative reconstructions differed only by L1 wavelet regularization factors, which were empirically optimized for the brain and the spinal cord (0.004 and 0.001, respectively). This flexibility is a fundamental benefit of under sampling-based acceleration (using a specific sampling pattern and reconstruction) compared to acceleration methods, which are more dependent on scanner specifications.

In this study, we performed MWI analysis with a non-negative least squares algorithm developed by Kumar *et al.* (*45*), which exploits 3D spatial correlations (in addition to commonly used temporal regularization) to improve the accuracy and noise robustness of the resulting quantitative metrics. To facilitate comparison with previous studies, which have not had access to this analysis, and to disentangle effects of the CALIPR framework versus spatially regularized analysis, the reproducibility analysis presented in this study was duplicated using temporal regularization only. These results, available in Supplementary Materials, confirmed that additional spatial regularization improves the precision of MWI metrics (mean COV: brain MWF, 3.2%; cord MWF, 3.0%; brain IET2, 0.26%, and cord IET2, 1.4%) compared to those generated with temporal regularization only (mean COV: brain MWF, 3.7%; cord MWF, 3.2%; brain IET2, 0.28%; and cord IET2, 2.0%). A comparison of the temporal and spatial analyses is shown in fig. S1 for the same subject and slice as Fig. 2.

As for any approach where data are under sampled and strong regularization or constraints are applied, it is essential to understand the underlying assumptions made by the CALIPR framework subspace constraint. We implicitly assume that: (i) a principal component basis can provide an accurate, compact representation of our data; (ii) this accurate, compact representation of our data can still be generated in the presence of incoherent noise or artifacts; and (iii) we can design a *k*-space sampling scheme such that the under sampling artifacts are incoherent.

In the Supplementary Materials, we provide results from simulations related to these assumptions. In fig. S2, we demonstrate that subspaces with only ~6 to 8 components manage to span the vast majority of signal features in our data, with negligible errors in the resulting images and signal evolutions. The principal component subspace does therefore provide a compact, accurate representation of multi-echo T_2_ relaxation for the case of ideal, low-noise data (assumption 1).

Figure S3 shows that the addition of independent, Gaussian-distributed noise to each TE of the original dataset has a negligible effect on these early subspace components. This result is intuitive, because the singular value decomposition used for subspace generation represents an expansion of the data in a coordinate system where the covariance matrix is diagonal. In fig. S4, we further demonstrate that a subspace generated from data with additional incoherent noise or artifacts still provides a compact, accurate representation of the multi-echo T_2_ relaxation data (assumption 2).

In fig. S5, we display the CALIPR framework *k*-space sampling scheme and data reconstructed with varying amounts of regularization for the same representative subject shown in Fig. 2 from the CALIPR brain MWI reproducibility experiments. The unregularized Fourier transform reconstruction images in fig. S5 show that the variable-density Poisson distribution sampling scheme, generated with a different random seed for each TE, results in under sampling artifacts that are both spatially and temporally incoherent (assumption 3).

We note that fig. S4 also demonstrates the clear tradeoff between noise and bias for different subspace sizes. Small subspace sizes have a strong denoising effect but introduce bias (they fail to contain some signal features). Large subspace sizes avoid introducing bias (because they contain all the true underlying signal features) but at the cost of failing to suppress incoherent noise and artifacts (which are contained in the later subspace components). For a subspace size of ~6 to 8, these simulations show unstructured image and signal residuals, suggesting that underlying signal features have been captured, and there is negligible evidence of bias. However, with ~6 to 8 subspace components, there is still a strong denoising effect (fig. S4), which should improve the accuracy and precision of the data.

On the basis of these simulations, the fixed subspace size of 12 used for CALIPR reconstructions throughout this work should provide a conservative, accurate representation of the data that will err on the side of capturing all signal features at the risk of retaining some noise artifacts (compared to use of a smaller subspace size). In fig. S6, we investigate the subspace representation of actual reconstructed data for the same representative subject shown in Fig. 2 from the CALIPR brain MWI reproducibility experiments.

Figure S6A shows the 12 reconstructed subspace coefficient images (with image intensities normalized to make all the coefficient images visible), and fig. S6B shows the contributions of each of these components toward the reconstructed signal. The increasingly unstructured spatial patterns and negligible amplitudes of contributions from later subspace components (especially *K* > 6) provide further evidence that the fixed subspace size of 12 accurately spans the signal feature space and that the inclusion of more components would tend to only increase noise contributions.

Our simulations echo the results of *Does* et al. (*34*), who recently showed that PCA denoising was extremely effective at reducing MWF root mean square error, by a factor of approximately 2 to 4, for simulated MWI data. On the basis of these results, they predicted that the precision of MWF values calculated from typical in vivo MWI data could be improved by a factor of about 3 (*34*). Our strong reproducibility results are due, in part, to the effects of PCA dimensionality reduction, which is expected to improve conditioning of the fundamentally ill-posed multicomponent T_2_ mapping problem. By incorporating a form of data driven PCA directly into the reconstruction itself, the CALIPR framework provides images and quantitative maps with intrinsically higher precision than conventional techniques.

Previous subspace constrained image reconstructions for quantitative MRI applications typically generated a single subspace using simulated signals, based on an assumed model of the data, and used it for all future reconstructions (*25*, *46*). Alternatively, the signal evolutions could be generated on the basis of simulations that used quantitative values from a reference acquisition (*27*) or could be taken from the reference acquisition itself. These approaches can provide an extremely compact representation, appropriate for conventional, qualitative imaging techniques, and avoid the need to generate a new subspace for each acquired dataset. However, generating the subspace from simulated or reference acquisition signal evolutions enforces a priori expectations of the signal. Signal characteristics that fall outside of these expectations will be excluded from the reconstructed images and subsequent quantitative maps, inducing a logical circularity bias where the reconstructed signal is explicitly constrained to lie within a subspace of the expected signal. This bias is of particular concern for quantitative MRI techniques in the presence of unique signal characteristics, for example, when imaging tissue with pathology (such as lesions in demyelinating diseases). The CALIPR framework mitigates this potential bias by using an adaptive subspace, created from the data itself after a preliminary reconstruction step. This adaptive subspace makes the CALIPR framework agnostic to expectations of the acquired signal, and, because the subspace creation is an automated process, it avoids the need to generate a new subspace when imaging fixed tissue, phantoms, or even different anatomy (such as spinal cord instead of brain). Previous advanced MRI techniques such as time-resolved dynamic MRI have used similar approaches, where reconstruction subspaces are generated from the acquired data (*47*).

In their study of PCA image denoising for multicomponent T_2_ mapping, *Does* et al. (*34*) showed that PCA denoising reduced noise by a factor of approximately 2.5 for the first echo image of MWI data, with even larger noise reductions for images later in the echo train where SNR is lower due to T_2_ decay. This effect is evident when comparing the CALIPR echo images (Fig. 4B) with those of GRASE (Fig. 4C); the CALIPR images have a noticeable less noisy appearance, especially visible for long TE images. The pronounced denoising effect at later echo times is also apparent in our simulation results (fig. S4), where later echo times show relatively lower image and signal evolution errors, especially for smaller subspace sizes where the denoising effect is stronger. This intrinsic denoising contributes to the high SNR appearance of the CALIPR images and quantitative maps. Ultimately, this denoising effect also contributes to the improved conspicuity of lesions and more subtle pathology demonstrated by CALIPR, especially for the MWF metric, which is based on short T_2_ components that are notoriously difficult to characterize.

Because the CALIPR echo images are of comparable quality to the conventional 3D T_2_w image, shown in Fig. 4A, acquisition of a separate anatomical T_2_w image may be rendered redundant for exams including CALIPR MWI. Furthermore, the CALIPR brain MWI acquisition provides images with 56 different T_2_ weightings. The very long TE images available from CALIPR MWI make tissue pathology appear extremely pronounced and would be difficult to acquire with sufficient SNR using conventional fast spin-echo techniques.

This study had several limitations worth considering. We did not compare CALIPR with an in vivo gold standard reference MWI scan. True gold standard acquisitions for high-resolution quantitative MRI techniques tend to be harrowed by motion artifacts related to their extremely long acquisition times (~3 hours to acquire a fully sampled brain MWI dataset using the CALIPR sequence). Attempts to acquire a smaller volume often suffer from artifacts related to outer volume signal for 3D acquisition or slice profile and magnetization transfer effects for 2D acquisition (*48*). Our gold standard reference acquired for a post-mortem fixed brain sample allowed us to develop, assess, and compare CALIPR without the influence of variation from extraneous factors such registration quality between scans, motion artifacts, or differing SNR due to variations in subject positioning.

There were some limitations to using a post-mortem fixed brain sample; despite the clear improvement shown using the CAPLIPR approach compared to conventional CS, some loss of detail and alterations were observed. This was, in fact, expected because, to ensure the comparison was as fair as possible, we used a set of fixed conditions to isolate the improvements made by use of the adaptive subspace constraint (i.e., not using the optimal CALIPR sampling pattern used later for the reproducibility, sensitivity, and multicenter experiments). Further, fixed brain MWI is a notoriously difficult experiment because the fixation process changes the relaxation times, resulting in an extremely short window of T_2_ < ~20 to 30 ms for the myelin water component (*4*). This means that the number of echoes with myelin water signal above the noise floor is much lower than for in vivo MWI, leading to pronounced under sampling effects for the short T_2_ myelin water signal. Overall, this intrinsic difficulty was an advantage for our validation work, as it provided a stringent testing ground for optimization of the sampling scheme and other parameters.

Comparing MWF and IET2 values from exams 1 and 2 in our reproducibility experiments, we found a small bias for MWF values in the posterior internal capsule (mean bias +0.6%, *P* = 0.005) and temporal WM (+0.4%, *P* = 0.04). However, given that they constitute two weakly significant results out of the 42 two-sided one-sample *t* tests performed (MWF and IET2 in 16 brain and 5 cord ROIs), these results are likely spurious and would not survive a correction for multiple comparisons.

Throughout this study, we used a fixed subspace size of 12 components based on empirical optimization. Tamir *et al.* (*27*) have provided a thorough explanation of how subspace constrained reconstruction modulates image noise and detailed the inherent tradeoff between introducing bias (increasing model error) for too small a subspace and failing to suppress noise (reducing model precision) for too large a subspace. We found that a subspace size of 8 performed well in most scenarios without clear evidence of bias, but given the intended nature of quantitative MRI techniques, we chose to err on the side of failing to suppress noise due to use of a relatively large subspace. The method used for determining the size of the subspace remains an open topic that can be addressed with a variety of approaches. Subspace size can be chosen based on a Marchenko-Pastur distribution from random matrix theory (*33*, *34*, *49*), chosen based on a normalized model error tolerance (*27*), or, as in this work, fixed at a specific value based on empirical optimization (*46*). Instead of explicitly constraining the reconstruction to a subspace of PCA components, the reconstruction could include the entire basis set but with additional regularization applied to that dimension to promote sparsity. Last, alternative approaches may be able to provide an even more compact sparse representation than PCA, for example, by learning a low-dimensional manifold representation of the data (*50*, *51*).

Limited availability of MWI techniques across different scanner vendors, software levels, and hardware specifications has substantially restricted the utility of MWI and has led to the development of pseudo-quantitative MRI techniques with inferior specificity for myelin (*52*–*54*). Although we leave a rigorous multivendor comparison for future studies, in the Supplementary Materials, we demonstrate an early implementation of CALIPR MWI for an additional MRI vendor (GE, shown in fig. S7). This proof of concept demonstrated that the CALIPR framework is relatively easy to translate between scanners with different software or hardware specifications. We are currently developing CALIPR MWI for a third high-field MRI manufacturer, with the goal of providing an openly available, robust framework capable of performing rapid, precise, sensitive MWI on the three largest vendors. To fully realize the potential of CALIPR MWI, further work is required to harmonize matched MWI sequences across scanner manufacturers and characterize the intersite reproducibility.

At the time of this study, the CALIPR framework is already being applied to study myelin content in the brain and spinal cord of subjects living with multiple sclerosis (~120 subjects). We have also acquired data in a large number of healthy participants to facilitate the creation of a normative atlas for use in future comparisons.

In conclusion, the CALIPR framework drastically reduces the acquisition time of MWI by acquiring a small fraction (~4%) of the dataset and then exploiting data redundancy to recover high-quality images. Intrinsic denoising properties contribute to the improved reconstruction performance compared to conventional CS MRI acceleration and the high precision found for CALIPR MWF and IET2 metrics in the healthy brain and spinal cord. In the context of demyelinating disease pathology (the hallmark application for quantitative MRI biomarkers of myelin), a combination of improved spatial and parametric resolution leads to markedly increased sensitivity to pathology for CALIPR compared to a current, widely used MWI technique. We implemented CALIPR for MWI of brain and cervical spinal cord and have demonstrated implementation for two of the three largest MRI manufacturers.

In addition to providing rapid, high-resolution MWI with improved precision and sensitivity to pathology, the inherent flexibility of this framework can be leveraged to bring similar benefits to other MRI techniques. In particular, this work could be similarly transformative for other quantitative MRI techniques where high dimensionality can be leveraged to reduce acquisition times and improve precision of the resulting metrics. By simultaneously improving data quality for research applications and reducing acquisition time for clinical applications, the CALIPR framework facilitates quantitative myelin imaging without compromise and negates the need to settle for acquiring less-informative MRI.

## MATERIALS AND METHODS

### Experimental design

This study was approved by the University of British Columbia Clinical Research Ethics Board. All volunteers provided written informed consent.

Validation, reproducibility, and sensitivity experiments were performed at 3.0 tesla on an Ingenia Elition X scanner (software version R5.7.1, Philips Healthcare, Best, The Netherlands). Brain imaging used a 32-channel head coil, while spinal cord imaging used a 16-channel head-neck coil with a 12-channel posterior spine coil.

### CALIPR acquisition

To allow for prospective under sampling with a user defined sampling scheme on Philips scanner software, we modified the University of Amsterdam Academic Medical Center (AMC) “PROspective Undersampling in multiple Dimensions” (PROUD) software patch (*55*, *56*) to allow for use with MESE pulse sequences. User defined sampling files were provided to specify which phase-slice encoding matrix data points to acquire at each TE in order. This provided complete flexibility to acquire any sampling on a cartesian grid.

**Fig. 7.**
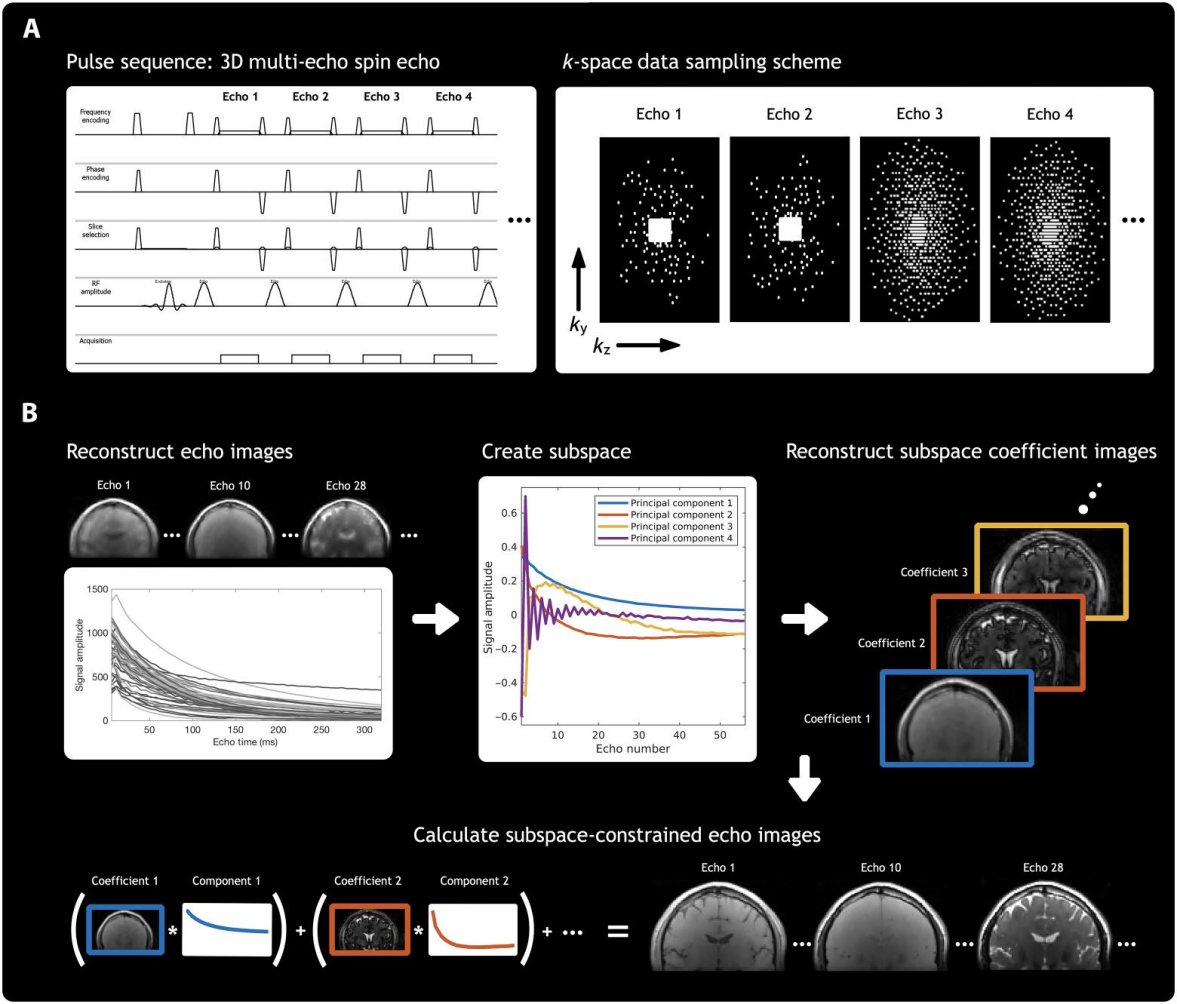
CALIPR framework acquisition and reconstruction. A graphical overview of the CALIPR framework methodology. For (**A**) acquisition and (**B**) reconstruction. For (A) image acquisition, the pulse sequence phase encoding gradients are modified to sample data incoherently across the additional nonspatial dimension present for many magnetic resonance imaging techniques. For (B) reconstruction, images from an initial naïve reconstruction are used to generate a subspace, calibrated for that specific dataset. A second and final reconstruction is then performed, explicitly constrained to this low-dimensional subspace. This combined acquisition and reconstruction framework exploits redundancy present in additional, nonspatial dimensions of the data by sampling in a way that makes under sampling artifacts less coherent and therefore more easily suppressed by a sparsity-promoting reconstruction, while true image features remain coherent and easily recoverable.

For our under sampling scheme, depicted in Fig. 7A and also in fig. S5, we used a temporally incoherent sampling scheme by generating a variable-density Poisson distribution with a different random seed for each TE. An elliptical *k*-space shutter was applied before the sampling distributions were projected onto a grid with a uniform under sampling factor of 2 in the phase direction. The uniform under sampling grid was used to effectively spread more of the incoherent aliasing artifacts across this direction of the imaging receive coils, as it tends to have a better *g* factor.

Fully sampled central calibration regions were acquired at the first two TEs to ensure sufficient characterization of stimulated echoes effects, which is essential for accurate estimation of refocusing flip angles and T_2_ relaxation components during MWI analysis (*57*–*59*). The central calibration region also allows for estimation of coil sensitivity maps directly from the data, making the acquisition self-contained (without the need for separate coil reference prescans).

Last, although these central calibration regions slightly reduce the overall incoherence of the sampling scheme, they also ensure that sufficient low-frequency *k*-space data are acquired to detect the short T_2_ relaxation components that are of paramount importance for MWI.

The sampling scheme was empirically optimized using retrospective under sampling reconstructions before final validation work was performed to verify performance in prospective under sampling conditions. Sampling schemes to be used for accelerated, prospectively under sampled acquisitions were generated offline in MATLAB (R2019b) for the appropriate matrix size.

### CALIPR reconstruction

The CALIPR reconstruction process outlined in Fig. 7B involves four steps:

1) This step is simply a conventional CS reconstruction of the dataset, without down sampling or otherwise modifying the data. It uses an iterative reconstruction with L1 wavelet spatial regularization to generate images at each echo time.

2) An intensity threshold is applied to mask out background noise regions, and then these multi-echo signals undergo singular value decomposition. This generates a basis set composed of principal components of the signal evolution across echo times. The data can now be represented in this basis of principal components instead of the original basis of echo times. The principal component basis is then truncated by keeping only some of the first components (12 components were used throughout this study). This truncated principal component basis is the subspace used in the next step as a reconstruction constraint.

3) A second iterative image reconstruction is performed, identical to that in step 1 but with an explicit subspace constraint. This iterative reconstruction also uses L1 wavelet spatial regularization but solves for coefficient images corresponding to amplitudes of each subspace component (rather than images at each echo time, as in step 1).

4) Last, images at each echo time are calculated from the results of step 3 by summing the contributions of the subspace coefficient images at each echo time. These echo time images can then be used for subsequent MWI analysis and calculation of quantitative metric maps.

The forward problem for the conventional CS reconstruction in step 1 can be written as

$$\underset{x}{\min}\frac{1}{2}{\left\|y-MFSx\right\|}_{2}^{2}+\lambda {\left\|T(x)\right\|}_{1}$$

for acquired data *y*, sampling mask *M*, Fourier transform *F*, coil sensitivity maps *S*, images at each echo time *x*, regularization factor λ, and wavelet transform *T*(*x*).

In step 2, singular value decomposition provides Φ, a basis set composed of signal evolution principal components. A low-dimensional subspace, Φ*_K_*, can be created by truncating this basis set, retaining only *K* principal basis components, where $x\approx {\text{Φ}}_{K}{\text{Φ}}_{K}^{H}x$.

In step 3, we reconstruct the data a second time but now enforcing an explicit subspace constraint by representing the data in a lifted space where we solve for *K* different subspace coefficient images, $\alpha ={\text{Φ}}_{K}^{H}x$. For the extended CS forward model in step 3, the reconstruction problem becomes

$$\underset{\alpha }{\min}\frac{1}{2}{\left\|y-MFS{\text{Φ}}_{K}\alpha \right\|}_{2}^{2}+\lambda {\left\|T(\alpha )\right\|}_{1}$$

for subspace Φ*_K_* and ultimately solving for subspace coefficient images α. The sparsity enforcing term (with wavelet transform *T*) remains unchanged except that it now operates on the subspace coefficient images instead of the echo time images. We refer the reader to the earlier Discussion section for an analysis of assumptions, caveats, and quality controls related to this subspace approximation.

Last, in step 4, images at each echo time are calculated from the subspace coefficient images as *x* = Φ*_K_*α.

Reconstructions were solved offline in MATLAB (R2019b) using FISTA (*60*) with the BART software package (*61*). Coil sensitivity maps were estimated from the echo 1 calibration region using ESPIRiT (*62*). To reduce reconstruction times (subspace reconstruction: ~12 min for the brain and ~8 min for the cord), *k*-space data were compressed to eight virtual channels (*63*) and reconstructions were solved on a graphics processing unit (GPU, NVIDIA Titan RTX).

In figs. S5 and S6, we show underlying details of the CALIPR framework reconstruction, both for the same representative subject shown in Fig. 2, from the CALIPR brain MWI reproducibility experiments. Figure S5 displays the sampling scheme and effects of reconstruction with different regularization, including an unregularized Fourier transform and coil combination where the incoherent under sampling artifacts are clearly visible.

Figure S6A provides a visualization of the 12 reconstructed subspace coefficient images, with image intensities normalized to make all coefficient images visible. In fig. S6B, the mean amplitude of each subspace coefficient image is plotted on a logarithmic scale. Note that the later subspace components (*K* > 6) have increasingly unstructured spatial patterns and amplitudes of contributions that are orders of magnitude smaller than early components, which suggests that the fixed subspace size of 12 should conservatively provide an accurate, low-error representation of signal features in each dataset.

### Myelin water imaging analysis

For MWI analysis, we used a modified version of the multicomponent T_2_ analysis algorithm previously introduced by Kumar *et al.* (*45*), which uses spatial and temporal regularization to iteratively refine the resulting flip angle map and T_2_ distributions. Compared to the version published previously, the analysis used here had relaxed criteria for the refinement of the spatial regularization factors and used fewer spatial analysis iterations (two instead of six).

### Validation experiments

A reference MWI sequence was developed using a post-mortem, single-hemisphere fixed brain sample donated by a subject with multiple sclerosis. The use of a fixed brain sample facilitated development, optimization, and validation in controlled conditions, without the influence of factors such as subject motion and image coregistration.

The reference acquisition was acquired with a 3D MESE sequence (field of view (FOV) of 240 mm by 192 by 100 mm, acquired resolution of 1.7 mm by 1.7 mm by 1.7 mm, reconstructed resolution of 1.5 mm by 1.5 mm by 1.5 mm, 56 echoes, ΔTE of 5.6 ms, TR of 1252 ms, and fully sampled acquisition time of 2 hours:47 min:25 s) in 2 hours:8 min:10 s with an extremely conservative CS under sampling acceleration factor of 1.3 (76.9% of the dataset).

Three reconstructions were performed and kept identical for comparison (iterative CS reconstruction, coil sensitivity maps, L1 wavelet regularization, etc) apart from the following differences:

1) The Reference version used the entire acquired dataset.

2) The accelerated CS version used data retrospectively under sampled by a spatially incoherent variable density Poisson distribution with an acceleration factor of 14.6 (6.8% of the dataset).

3) The accelerated CALIPR version used data retrospectively under sampled by a spatially and temporally incoherent variable density Poisson distribution with the same acceleration factor (14.6, 6.8% of the dataset) and the addition of the CALIPR subspace constraint.

The resulting echo images were analyzed using parameters appropriate for fixed brain, and MWF maps were generated for comparison.

### Reproducibility experiments

We implemented CALIPR for in vivo MWI of the brain and cervical spinal cord.

The brain acquisition used a slightly modified version of the aforementioned fixed brain reference sequence (3D MESE, FOV of 240 mm by 200 mm by 100 mm, acquired resolution of 1.7 mm by 1.7 mm by 1.7 mm, reconstructed resolution of 1.0 mm by 1.0 mm by 1.0 mm, 56 echoes, ΔTE of 6.0 ms, TR of 1252 ms, and fully sampled acquisition time of 2 hours:57 min:20 s) acquired in 7 min:26 s with an under sampling acceleration factor of 23.9 (4.2% of the dataset).

The spinal cord acquisition used a 3D MESE sequence (FOV of 180 mm by 152 mm by 60 mm, acquired resolution of 1.0 mm by 1.0 mm by 5.0 mm, reconstructed resolution of 0.62 mm by 0.62 mm by 2.5 mm, 48 echoes, ΔTE of 8.0 ms, TR of 1120 ms, and fully sampled acquisition time of 45 min:27 s) and was acquired in 8 min:23 s with an under sampling acceleration factor of 5.4 (18.4% of the dataset). The cord imaging FOV was centered at the C3/C4 level of the cervical spinal cord and angled so that slices were perpendicular to the cord.

Five healthy participants without documented history of brain or spinal cord disease or injury participated in the study (four male, median age of 27 years, range 23 to 33 years). Anatomical images were acquired for each subject to use for ROI segmentation and as an anchor image space during analysis: a sagittal 3D T_1_-weighted magnetization-prepared rapid gradient-echo image (T_1_w) for the brain and an axial 2D multislice T_2_*-weighted multi-echo gradient echo image (T_2_*w) for cord.

For both brain and cord, each CALIPR MWI acquisition was performed twice, in separate exams with repositioning (a total of four exams). Advanced Normalization Tools, Spinal Cord Toolbox (SCT), and FMRIB Software Library tools were used for image analysis (*64*–*66*).

For brain, T_1_w and MWI echo 1 images underwent N4 bias field correction for low-frequency intensity nonuniformities (*67*). Tissue segmentations were initialized by registration with the OASIS template and priors (*68*) and then refined using Atropos *n*-tissue segmentation (*69*). Images were masked to brain only before MWI echo 1 images were rigidly aligned with the corresponding subject’s T_1_w image.

T_1_w images were registered to a template created in-house using data from 100 healthy volunteers (*70*). Optimized ROIs were generated using the probabilistic joint label fusion framework (*71*) and warped to each subject’s T_1_w image space. Brain ROIs include all WM, all GM, and combined WM and GM from T_1_w image segmentations, along with nine additional WM ROIs [all Johns Hopkins University (JHU) WM labels combined: genu of CC; splenium of CC; whole CC; posterior internal capsule; and frontal, occipital, parietal, and temporal lobes masked to WM) and four additional GM ROIs (cortical GM, caudate, thalamus, and putamen).

For the spinal cord, we produced cord and cerebrospinal fluid (CSF) segmentations for the T_2_*w and MWI echo (final TE) images (*72*, *73*), as well as GM segmentations for the T_2_*w images (*74*). For each subject, images were masked to cord and CSF only before the MWI final echo image was rigidly aligned with the corresponding subject’s T_2_*w image.

T_2_*w images were registered with the T_2_*-weighted SCT PAM50 template (*75*), centered at the C3/C4 level of the cord, and registrations were refined using the T_2_*w GM segmentation to improve alignment of intracord structure. To reduce the influence of partial volume effects, the resulting probabilistic cord ROIs were thresholded to only include voxels with probability >0.5. Spinal cord ROIs include the WC, WM, GM, DC, and LCSTs.

We quantified results within each ROI by their median to account for the skewed distribution of metric values within ROIs, and we chose similar ROIs and statistical measures to previous studies to facilitate comparisons (*18*, *41*, *76*, *77*). MWF and IET2 results from exams 1 and 2 were compared using Bland-Altman plots. The 95% limits of agreement, RCs, COVs, and ICCs were calculated as in recent MWI studies (*18*, *19*). For MWF and IET2 in each ROI, a two-sided one-sample *t* test was performed on the difference between exams 1 and 2 to test for significant biases.

### Sensitivity experiments

We also aimed to assess the sensitivity of CALIPR MWI to pathological tissue changes and compare the sensitivity to a common, currently adopted MWI technique. To that end, we acquired data from a subject living with clinically definite relapsing-remitting multiple sclerosis fulfilling the 2017 revised MacDonald criteria for diagnosis (*78*) [male, age 60 years, expanded disability status scale of 2.0 (*35*), and disease duration of 12 years].

The imaging protocol included typical anatomical imaging for a multiple sclerosis exam: T_1_w, T_2_w, and T_2_w FLAIR, and proton density–weighted images acquired with 3D fast spin-echo sequences.

CALIPR MWI brain data were acquired as described in the “Reproducibility experiments” section. For comparison, we also acquired a commonly used 3D multi-echo GRASE sequence (FOV of 230 mm by 192 mm by 100 mm, acquired resolution of 1.0 mm by 2.0 mm by 5.0 mm, reconstructed resolution of 1.0 mm by 1.0 mm by 2.5 mm, 48 echoes, ΔTE of 8.0 ms, TR of 1079 ms, and fully sampled acquisition time of 44 min:55 s) acquired in 6 min:10 s with echo planar imaging (EPI) factor of 3 and parallel imaging under sampling acceleration factor of 2.5 (40.0% of the dataset) for a total acceleration factor of 7.5.

CALIPR and GRASE MWI data were processed as described in the “Reproducibility experiments” section. Echo images were shown in addition to MWF and IET2 maps for comparison of source MWI data quality. All results are shown for data aligned in T_1_w image space to ensure that the exact same anatomical locations were being compared.
