# Supplementary material for: The CALIPR framework for highly accelerated myelin water imaging with improved precision and sensitivity
Source: Sci Adv. 2023 Nov 1;9(44):eadh9853. doi: 10.1126/sciadv.adh9853 (PMC10619933; doi:10.1126/sciadv.adh9853)
Supplement: Supplementary file 1 — Supplementary Text Figs. S1 to S7 Tables S1 and S2 [file sciadv.adh9853_sm.pdf]

Science Advances
AAAS

Supplementary Materials for

The CALIPR framework for highly accelerated myelin water imaging with improved precision and sensitivity

Adam V. Dvorak *et al.*

Corresponding author: Adam V. Dvorak, adam.dvorak@ubc.ca

Sci. Adv. **9**, eadh9853 (2023)
DOI: 10.1126/sciadv.adh9853

This PDF file includes:

Supplementary Text
Figs. S1 to S7
Tables S1 and S2

Supplementary Text

CALIPR IET2 Reproducibility in Healthy Brain and Spinal Cord

The CALIPR IET2 reproducibility results are summarized here in **Table S1** for brain and **Table S2** for spinal cord. The CALIPR IET2 mean RC, COV, and ICC for brain were 0.05, 0.26 %, and 0.98, respectively, and for spinal cord were 0.35, 1.4 %, and 0.74, respectively.

CALIPR Data Analyzed with Temporal Regularization Only

In this work, we performed myelin water imaging (MWI) analysis using a modified version of a non-negative least squares algorithm originally developed by *Kumar et al.*. This algorithm exploits 3D spatial correlations (in addition to the commonly-used temporal regularization) to improve accuracy and noise robustness of the estimated flip angle error map and calculated T_2 distribution, ultimately improving the resulting quantitative metrics (45).
Here we used a modified version, which relaxed the criteria for optimization of the spatial regularization factors and ran fewer iterations (2 instead of 6) to refine the results. These changes drastically reduced the processing time but had a negligible effect on the quantitative maps. To facilitate comparison with previous studies which have not had access to this analysis, and to disentangle effects of the CALIPR framework versus spatially regularized analysis, the reproducibility results presented in this study were duplicated using conventional MWI analysis with temporal regularization only.

Figure S1 shows individual slices of CALIPR quantitative maps from the same exam, calculated using each analysis, along with voxel-wise difference maps between exams 1 and 2, for each analysis. Although differences in the maps are visually subtle, the reproducibility difference maps (**Figure S1**) show reduced extent and magnitude of differences between exams 1 and 2 for analysis with spatial regularization.

For reproducibility of CALIPR metrics calculated using analysis with temporal regularization only, MWF mean repeatability coefficient (RC), coefficient of variation (COV), and intra-class correlation coefficient (ICC) were 0.8, 3.7 %, and 0.92 for brain and 2.3, 3.2 %, and 0.85 for spinal cord. IET2 mean RC, COV, and ICC were 0.06, 0.28 %, and 0.97 for brain and 0.50, 2.0 %, and 0.69 for spinal cord.

When considered together, these results confirm that additional spatial regularization improves the precision of MWI metrics (mean COV: brain MWF 3.2 %, cord MWF 3.0 %, brain IET2 0.26 %, cord IET2 1.4 %), compared to those generated with temporal regularization only (mean COV: brain MWF 3.7 %, cord MWF 3.2 %, brain IET2 0.28 %, cord IET2 2.0 %).

It is worth noting that the CALIPR reproducibility metrics for data analyzed with temporal regularization only (without the use of 3D spatial correlations) still compare favourably to the literature MWI reproducibility metrics presented in the manuscript **Discussion**. Comparing reproducibility metrics between the two analyses confirms that the strong CALIPR reproducibility is mainly due to the intrinsic precision of the reconstruction. This intrinsic precision is supplemented by use of MWI analysis with 3D spatial correlations, not caused by it.

<u>Simulations to Characterize the CALIPR Framework Subspace Constraint</u>

In this section, we present results from simulations performed to validate the assumptions made by the CALIPR framework approach.

A highly sampled MWI dataset was acquired in 2h:2m:40s for a single healthy volunteer (male, 25 years) with a 3D MESE sequence (FOV 230x192x100mm^3, acquired resolution 1.0x2.0x2.0mm^3, reconstructed resolution 1.0x1.0x1.0mm^3, 56 echoes, ΔTE 5.7 ms, TR 1277 ms). This acquisition was performed at 3.0 Tesla on an Ingenia Elition X scanner (software version R5.7.1, Philips Healthcare, Best, the Netherlands) using a 32-channel head coil. The conventional product reconstruction was performed to generate echo time images, which were exported from the scanner.

As in the CALIPR reconstruction process, an intensity threshold is applied to mask out noise regions before the remaining signals undergo singular value decomposition to generate a temporal basis composed of principal components of the signal evolution across TEs. The accuracy of a range of subspace sizes can be assessed by projecting data into the subspace representation (whereby only signal features present in the subspace are retained), calculating the corresponding data in the original basis of TE times, then comparing the resulting TE data (after projection) with the original data.

We performed this investigation first for the case of highly sampled, low-noise data using the original unmodified magnitude dataset (**Figure S2**).

We also investigated the case of noisy data, by adding independent, gaussian distributed noise to each TE of the original dataset. Echo images from the original and noisy datasets are shown in **Figure S3**, along with plots of the first 6 subspace components generated from each.

The accuracy of a range of subspace sizes is shown in **Figure S4** for the noisy data (using the subspace generated from the noisy data), with comparison to the original, unmodified data.

Multi-Vendor Proof-of-Concept

In this section, we implemented CALIPR brain MWI on an additional MRI scanner vendor to demonstrate the relative ease of translating this framework to different techniques or platforms.

We implemented CALIPR for an additional MRI vendor (GE, whereas previous data were acquired using Philips) and performed experiments at 3.0 Tesla on a Discovery MR750 scanner (software version DV26.0_R03, GE Healthcare, Waukesha, WI) using a 32-channel head coil. The GE Healthcare scanner software was modified to allow user defined sampling for a variety of multi-echo pulse sequence acquisitions so that, similarly to the Philips system, a user defined sampling file could be provided to flexibly specify which phase encoding matrix data points to acquire on a cartesian grid.

The GE brain acquisition was similar to the brain acquisition described in the manuscript **Materials and Methods: Reproducibility Experiments** (3D MESE sequence, FOV 240x192x100mm^3, acquired resolution 2.0x2.0x2.0mm^3, reconstructed resolution 1.5x1.5x1.5mm^3, 56 echoes, ΔTE 7.168 ms, TR 1804 ms, fully sampled acquisition time 2h:56m:04s) and was acquired in 10m:39s with an under sampling acceleration factor of 16.5 (6.0% of the dataset). Lower acquired resolution was used to offset additional acquisition time caused by the longer TR, due to differences in radiofrequency pulses and specific absorption rate limitations.

We acquired CALIPR brain MWI for a single healthy subject (female, 27 years) and processed the data as described in the manuscript **Materials and Methods: Reproducibility Experiments**.

Individual slices of CALIPR MWF and IET2 maps are shown in **Figure S7**.

The CALIPR framework accelerates acquisition by collecting a smaller amount of data, rather than by collecting data faster, meaning that it can be relatively easily generalized to imaging techniques with different pulse sequences as well as translated between scanners with different software or hardware specifications.

Future work is required to optimize this implementation, harmonize matched MWI sequences across MRI scanner manufacturers, and ultimately characterize the inter-site reproducibility.

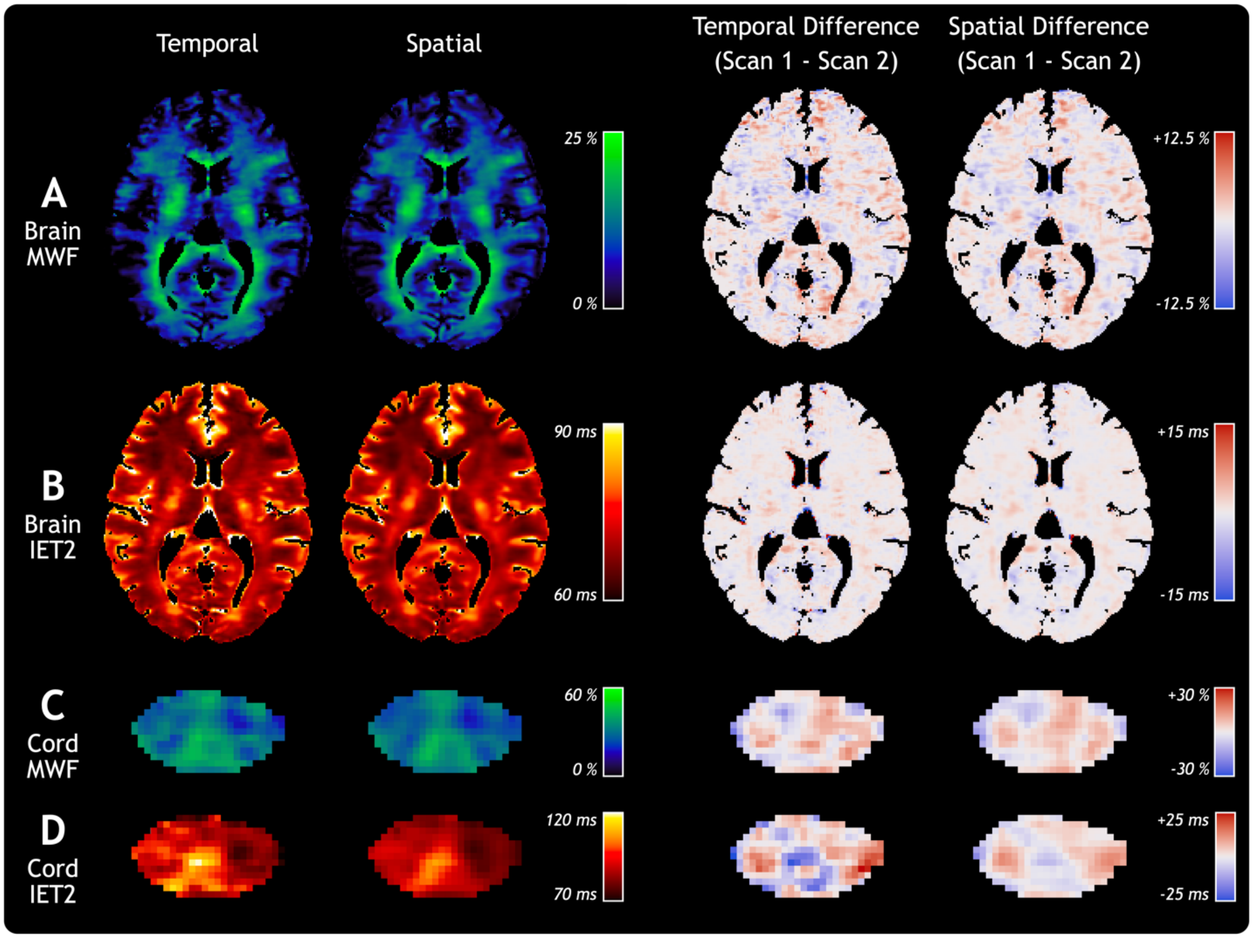

Fig. S1. Comparison of myelin water imaging metrics in brain and spinal cord calculated using analysis with temporal regularization only versus additional spatial regularization. Individual slices of (**A**) brain myelin water fraction (MWF), (**B**) brain geometric mean of intra/extra-cellular T_2 (IET2), (**C**) spinal cord MWF, and (**D**) spinal cord IET2 for a single healthy subject are shown aligned in the subject's structural image space (T_1-weighted for brain, T_2*-weighted for cord). From left to right, columns show results from the same CALIPR myelin water imaging exam for each analysis and the voxel-wise difference maps between exams 1 and 2 for each analysis.

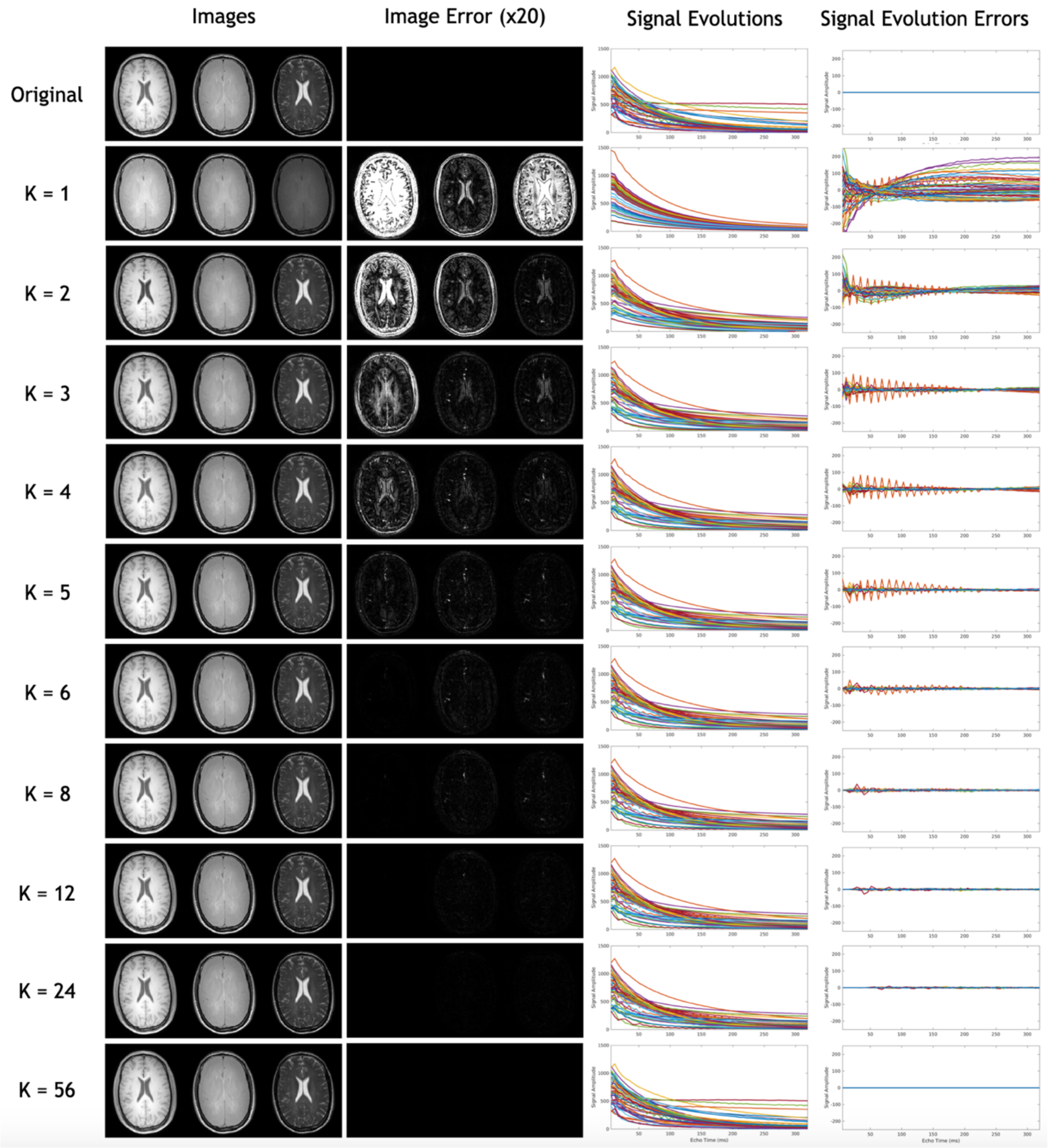

Fig. S2. Compact, low-error subspace representation of multi-echo T_2 relaxation for the case of ideal, low-noise data. From left to right, columns show echo time images (echoes 1, 10, and 28), image residuals compared to the original data, signal evolutions, and signal evolution residuals compared to the original data. From top to bottom, rows depict results for data compressed into subspace representations of the data with a range of subspace sizes. Note that the image errors are magnified by a factor of 20 and that the signal evolutions are plotted with different y-axes than the signal evolution errors.

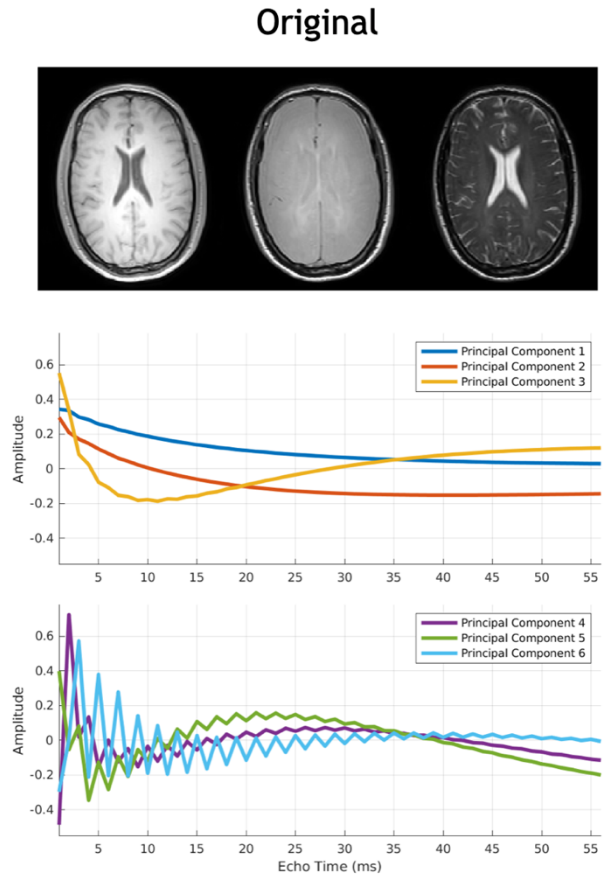

Fig. S3. Accurate, low-error subspace generation despite the addition of incoherent noise artifacts. Echo time images (echoes 1, 10, and 28) and the first 6 subspace components generated from the data are shown for (left) the case of ideal, low-noise data and (right) the case of highly noisy data. Note that the subspace components are nearly identical, despite the substantial different in incoherent artifacts between the two datasets.

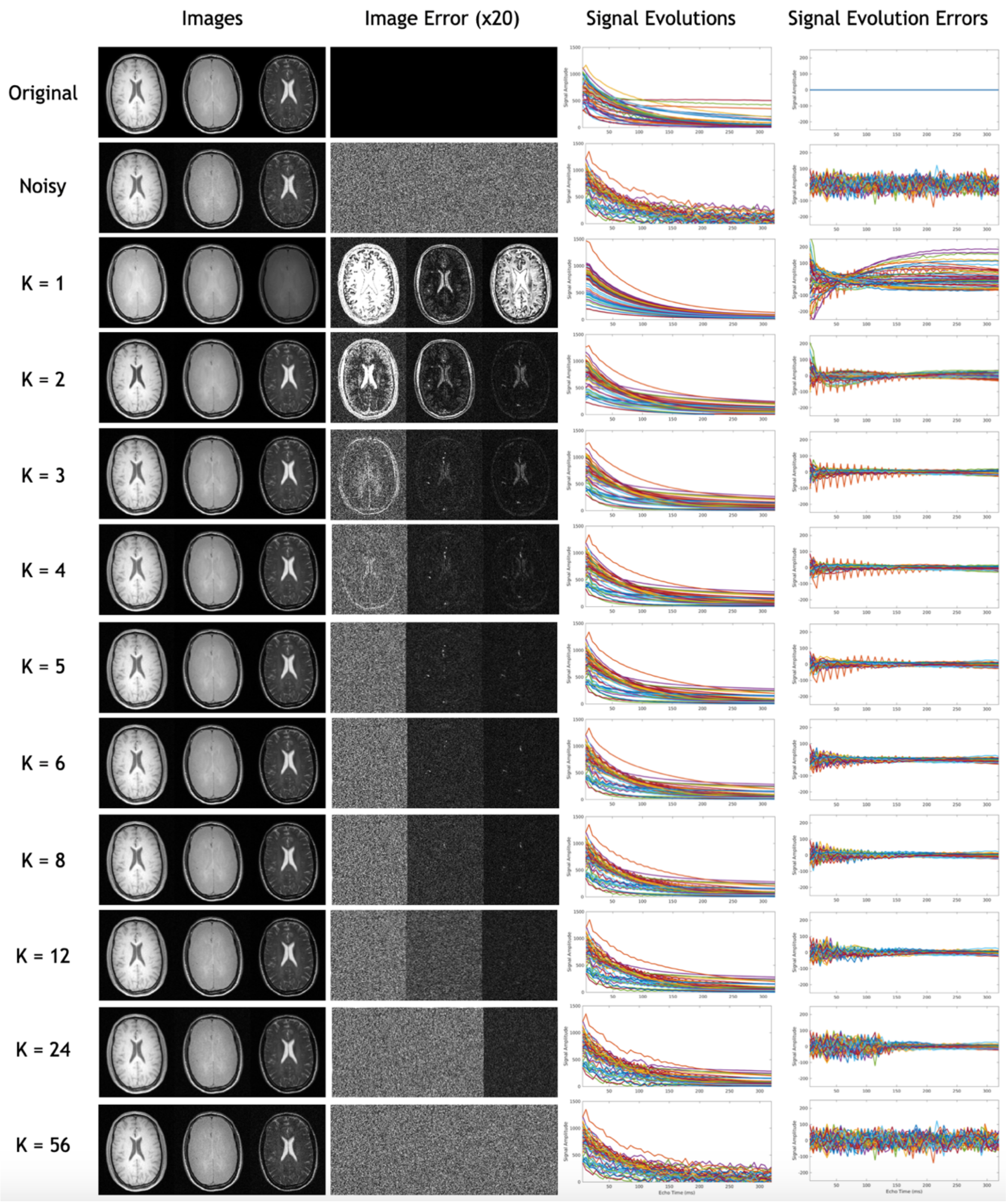

Fig. S4. Compact, low-error subspace representation of multi-echo T_2 relaxation for the case of highly noisy data. From left to right, columns show echo time images (echoes 1, 10, and 28), image residuals compared to the original data, signal evolutions, and signal evolution residuals compared to the original data. From top to bottom, rows depict results for data compressed into subspace representations of the data with a range of subspace sizes. Note that the image errors are magnified by a factor of 20 and that the signal evolutions are plotted with different y-axes than the signal evolution errors.

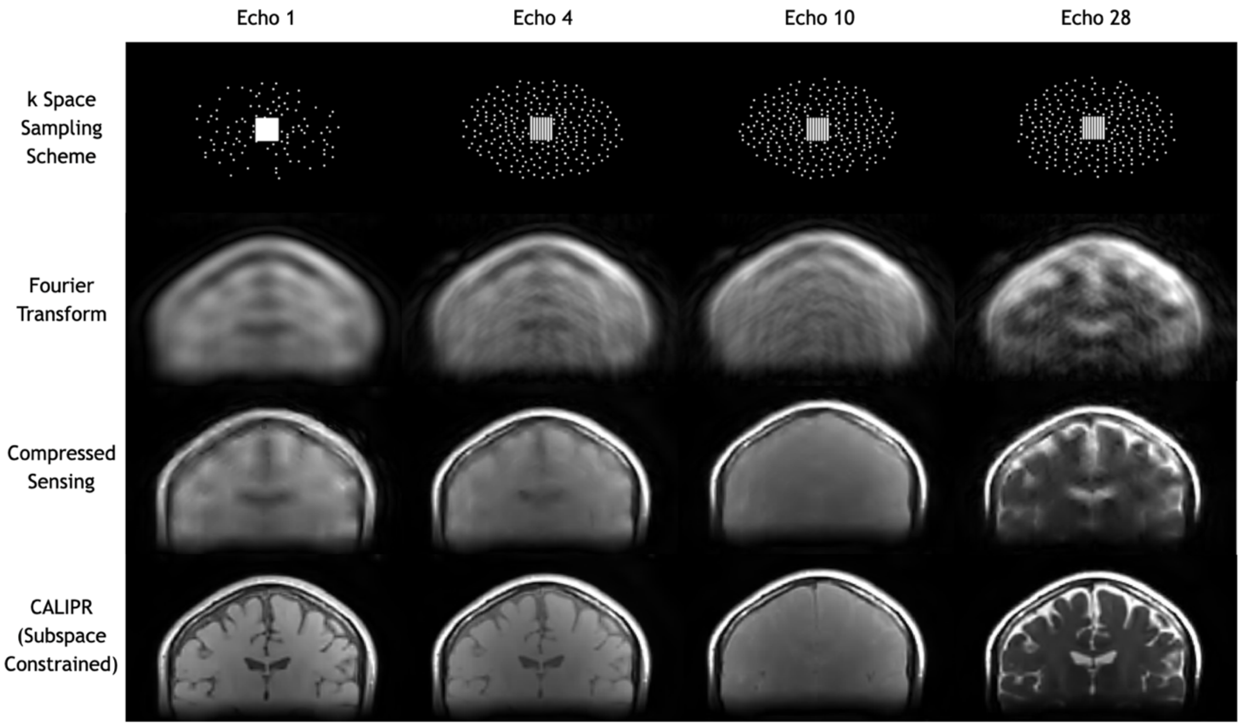

Fig. S5. Underlying details of CALIPR framework: Sampling scheme, incoherent under sampling artifacts, effects of reconstructions with different regularization. The incoherent k-space sampling scheme is depicted for echoes 1, 4, 10, and 28. The resulting incoherent under sampling artifacts are clearly visible for images generated using a conventional Fourier transform reconstruction. For reconstruction with spatial regularization (compressed sensing), the incoherent artifacts are blurred throughout the images. For reconstruction with spatial and temporal regularization (CALIPR, subspace constrained), true underlying signal features are recovered by the sparse subspace representation, while non-sparse incoherent artifacts and noise are suppressed. All images shown here are for the same representative subject shown in Figure 2, from the CALIPR brain MWI reproducibility experiments.

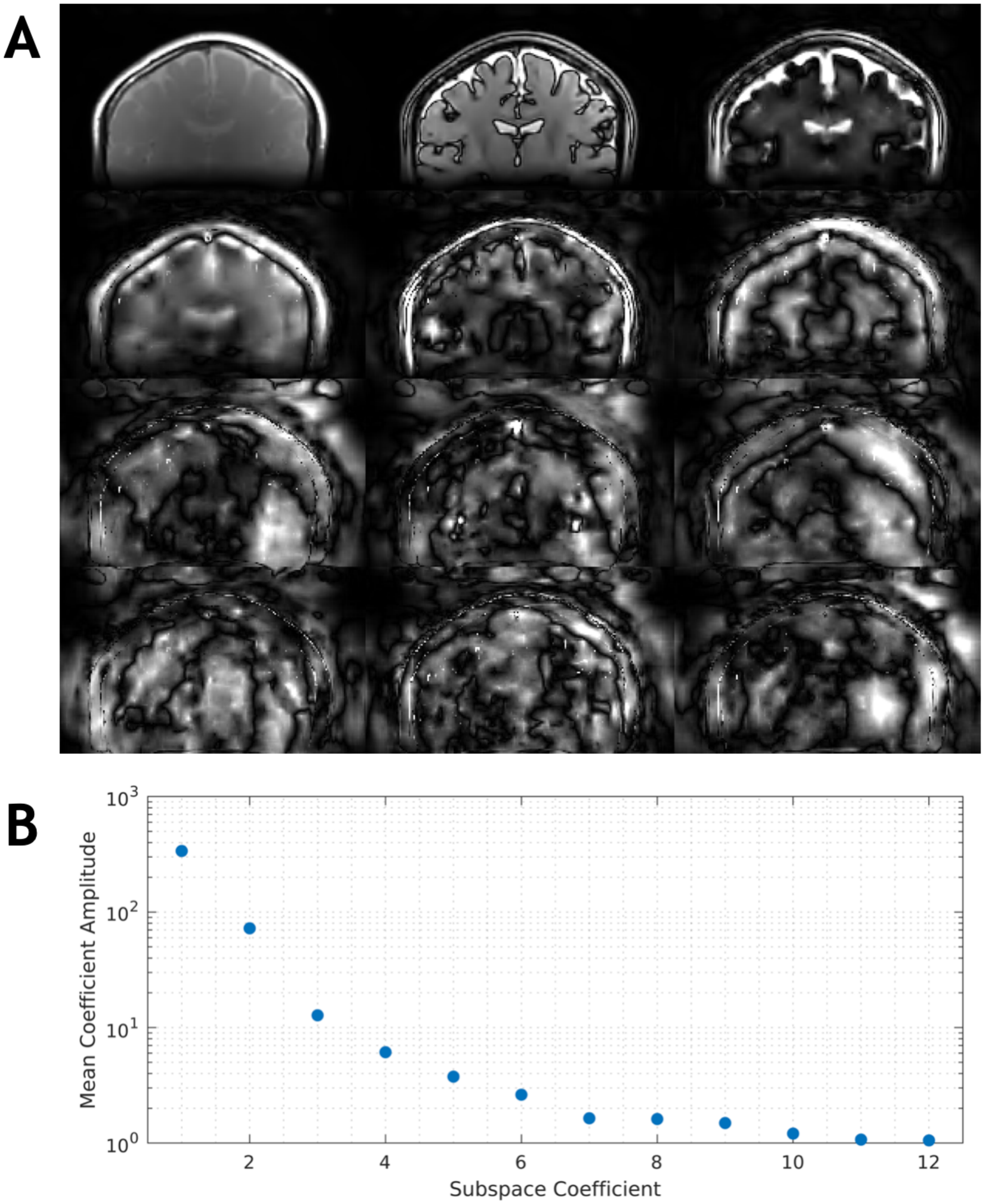

Fig. S6. Underlying details of CALIPR framework: Compact, accurate subspace representation demonstrated during reconstruction of prospectively under sampled in-vivo CALIPR data. (**A**) The 12 reconstructed subspace coefficient images are shown. Note that the image intensities were normalized to make all coefficient images visible. In (**B**), the mean amplitude of each subspace coefficient image is plotted on a logarithmic scale. The unstructured spatial patterns and negligible amplitudes of contributions from later subspace components suggest that the fixed subspace size of 12 should provide a conservative, accurate representation of the data. All images shown here are for the same representative subject shown in Figure 2, from the CALIPR brain MWI reproducibility experiments.

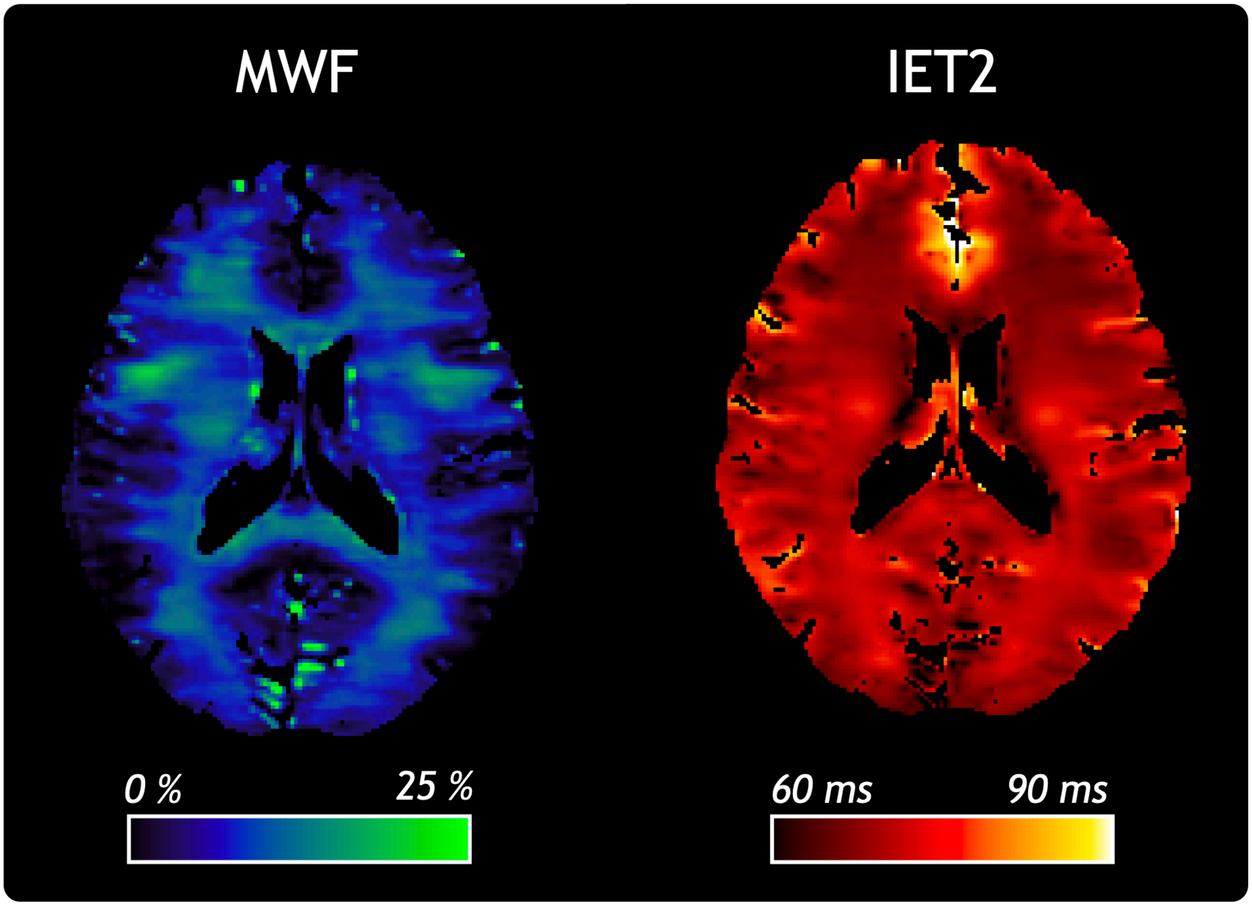

Fig. S7. Multi-vendor CALIPR myelin water imaging proof-of-concept. Individual slices of myelin water fraction (MWF) and geometric mean of intra/extra-cellular T_2 (IET2) are shown for a single healthy subject. The exam was performed using an early implementation of CALIPR myelin water imaging on a 3.0 Tesla scanner from an alternative vendor (GE Healthcare).

		CALIPR IET2 (*ms*)					
	ROI	Scan 1	Scan 2	LOA	RC	COV (%)	ICC
	WM&GM	73.8 ± 1.4	73.6 ± 1.5	-0.5 | 1.0	0.04	0.19	0.98
White Matter Regions	WM	72.5 ± 1.7	72.2 ± 1.8	-0.5 | 1.0	0.04	0.19	0.98
	All JHU	74.4 ± 1.7	74.0 ± 1.9	-0.5 | 1.4	0.06	0.31	0.97
	Genu	68.0 ± 2.4	67.8 ± 2.5	-0.5 | 1.0	0.05	0.29	0.99
	Splenium	75.0 ± 1.7	74.4 ± 1.5	-0.4 | 1.7	0.10	0.47	0.94
	Whole CC	72.9 ± 1.8	72.4 ± 1.7	-0.3 | 1.3	0.07	0.35	0.97
	Posterior Internal Capsule	77.7 ± 2.5	76.8 ± 2.4	-0.4 | 2.3	0.14	0.64	0.95
Lobe White Matter	Frontal	71.7 ± 1.9	71.6 ± 2.0	-0.7 | 1.0	0.04	0.20	0.99
	Occipital	72.4 ± 1.5	72.2 ± 1.7	-0.8 | 1.2	0.05	0.26	0.97
	Parietal	72.7 ± 1.6	72.4 ± 1.6	-0.5 | 1.0	0.04	0.18	0.98
	Temporal	71.0 ± 1.5	70.8 ± 1.5	-0.4 | 0.7	0.03	0.17	0.99
Gray Matter Regions	GM	75.5 ± 1.2	75.3 ± 1.2	-0.4 | 0.8	0.03	0.16	0.98
	Cortical	76.4 ± 1.3	76.2 ± 1.3	-0.4 | 0.8	0.03	0.16	0.98
	Caudate	70.0 ± 1.6	69.7 ± 1.5	-0.2 | 0.6	0.03	0.16	0.99
	Thalamus	71.2 ± 1.4	70.8 ± 1.5	-0.3 | 1.2	0.06	0.30	0.97
	Putamen	69.6 ± 2.0	69.4 ± 1.9	-0.2 | 0.7	0.04	0.20	0.99
Mean		72.8 ± 1.7	72.5 ± 1.7	-0.4 | 1.1	0.05	0.26	0.98

Table S1. CALIPR brain geometric mean of intra/extra-cellular T_2 (IET2) reproducibility. Reproducibility results for brain IET2 from repeated CALIPR scans, acquired in separate exams, for 5 healthy subjects. Mean and standard deviation of the region of interest (ROI) values are shown along with the following reproducibility metrics: the 95% limits of agreement (LOA negative|LOA positive), repeatability coefficients (RC), coefficients of variation (COV), and intra-class correlation coefficients (ICC). Brain ROIs include all white matter (WM), all gray matter (GM), and combined WM and GM from T_1-weighted image segmentations, along with nine additional WM ROIs (all JHU white matter labels combined, genu of corpus callosum (CC), splenium of CC, whole CC, posterior internal capsule, and frontal, occipital, parietal, and temporal lobes masked to WM), and 4 additional GM ROIs (cortical GM, caudate, thalamus, and putamen).

	CALIPR IET2 (*ms*)					
ROI	Scan 1	Scan 2	LOA	RC	COV (%)	ICC
WC	87.7 ± 2.0	87.2 ± 1.6	-2.5 | 3.6	0.18	0.8	0.82
WM	89.6 ± 2.5	88.9 ± 2.1	-2.5 | 4.1	0.21	0.9	0.87
GM	82.3 ± 1.9	82.5 ± 1.2	-4.7 | 4.3	0.30	1.3	0.53
DC	94.3 ± 5.3	91.9 ± 1.7	-5.9 | 10.8	0.45	1.7	0.81
LCST	91.2 ± 2.0	93.0 ± 4.9	-10.1 | 6.4	0.59	2.3	0.68
Mean	89.0 ± 2.7	88.7 ± 2.3	-5.1 | 5.8	0.35	1.4	0.74

Table S2. CALIPR spinal cord geometric mean of intra/extra-cellular T_2 (IET2) reproducibility. Reproducibility results for spinal cord IET2 from repeated CALIPR scans, acquired in separate exams, for 5 healthy subjects. Mean and standard deviation of the region of interest (ROI) values are shown along with the following reproducibility metrics: the 95% limits of agreement (LOA negative|LOA positive), repeatability coefficients (RC), coefficients of variation (COV), and intra-class correlation coefficients (ICC). Spinal cord ROIs include the whole cord (WC), white matter (WM), gray matter (GM), dorsal column (DC), and lateral corticospinal tracts (LCST).
